# A human iPSC-based perfusable and membrane-free neurovascular unit-on-chip connecting brain organoids to the blood–brain barrier

**DOI:** 10.1039/d6lc00157b

**Published:** 2026-09-11

**Authors:** Henrique Nogueira Pinto, Lois Kistemaker, Mikhail Ponomarenko, Arthur A. Ermakov, Manon Karsten-van Diepen, Susanne M. A. van der Pol, R. Jeroen Pasterkamp, Stephanie D. Beekhuis-Hoekstra, Nienke M. de Wit, Emma J. van Bodegraven, Helga E. de Vries, Elly M. Hol

**Affiliations:** a Amsterdam UMC location Vrije Universiteit Amsterdam, Department of Molecular Cell Biology and Immunology Amsterdam Netherlands he.devries@amsterdamumc.nl; b Amsterdam Neuroscience, Neuroinfection & inflammation Amsterdam Netherlands; c Amsterdam Infection & Immunity, Immunology Amsterdam The Netherlands; d Department of Translational Neuroscience, University Medical Center Utrecht, Utrecht University Utrecht Netherlands E.M.Hol-2@umcutrecht.nl; e Pimbio B.V. 's Hertogenbosch The Netherlands; f MS Center Amsterdam, Amsterdam UMC Location VU Medical Center Amsterdam The Netherlands

## Abstract

The blood–brain barrier (BBB) protects the central nervous system by restricting entry of harmful blood-borne factors, but this selectivity actively limits delivery of therapeutics to the brain. Because BBB function is shaped by dynamic interactions within the neurovascular unit (NVU), particularly between brain endothelial cells, pericytes, and astrocytes, there is a need for human-relevant models that capture both barrier properties and neurovascular crosstalk in a controlled setting. Here, we present a novel human induced pluripotent stem cell (iPSC)-based NVU-on-chip that couples a perfusable, vessel-like BBB compartment to an adjacent open-top chamber housing a human brain organoid (hBO) *via* a membrane-free, hydrogel-based interface. This design enables co-culture of BBB and parenchymal components in close proximity while allowing direct cell–cell interactions at the barrier–tissue boundary without an artificial porous membrane. Using this platform, we demonstrate stable on-chip co-culture and show that hBO-derived astrocytes can sprout, migrate toward the BBB compartment and establish direct contact with endothelial cells, recapitulating the NVU structure. This system offers a human-specific framework for modeling NVU biology in health and disease and for evaluating BBB penetration together with downstream effects of candidate therapeutics on hBO.

## Abstract 2

The CONNECT chip links a perfusable human blood–brain barrier to brain organoids without a porous membrane, enabling direct neurovascular interactions and simultaneous assessment of barrier function and brain responses.

## Graphical abstract

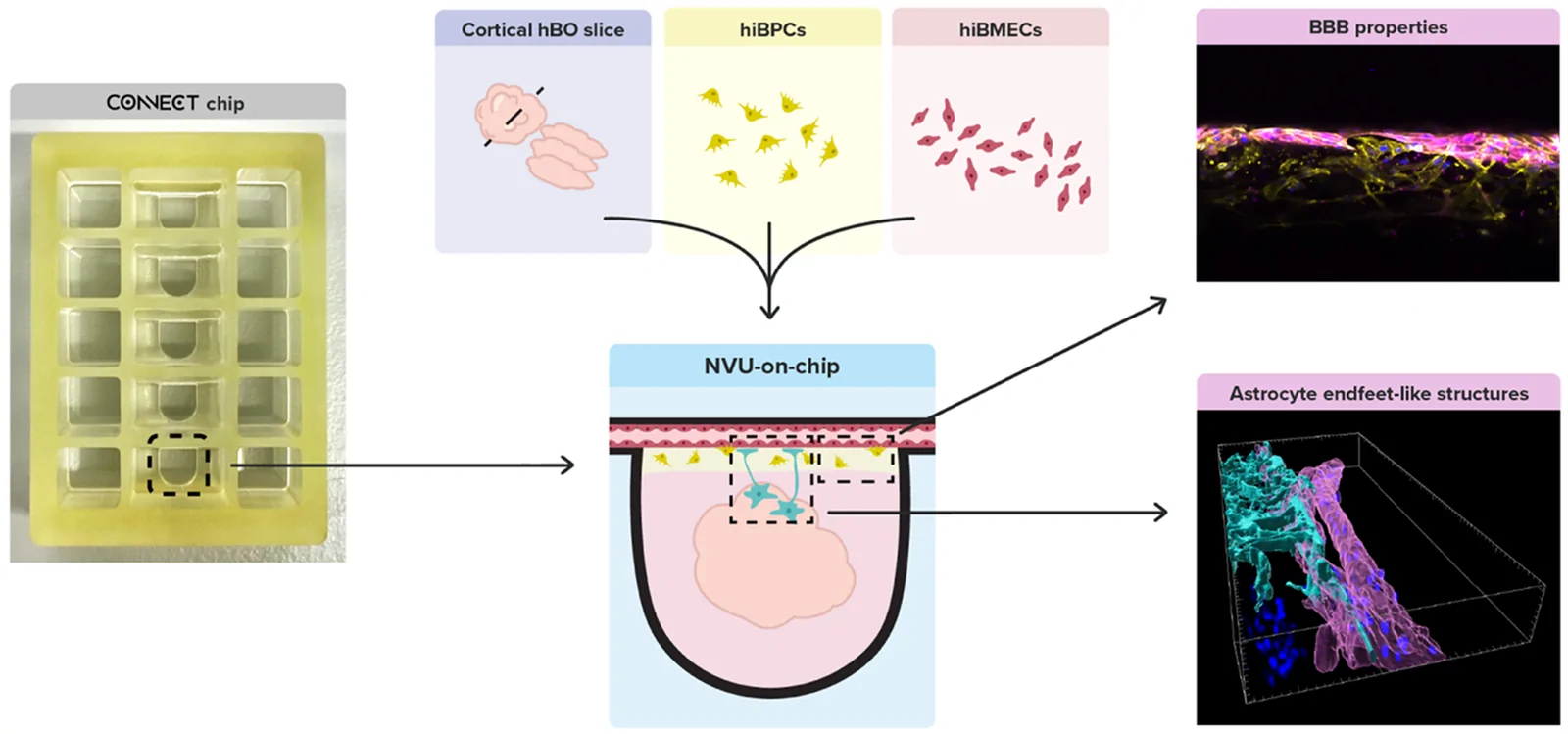

## 1. Introduction

Effective therapies for the treatment of many neurodegenerative diseases currently remain unavailable. The development of such therapies is hampered not only by a lack of understanding of disease mechanisms but also by the difficulty of testing if and how potential therapeutic compounds enter and affect the brain. What enters the brain from the periphery is strictly regulated by the blood–brain barrier (BBB).

The BBB is a cellular structure composed of brain microvascular endothelial cells (BMECs) and supported in its barrier function by pericytes and astrocytic endfeet.^1^ Whilst the BBB prevents the entry of harmful blood-borne substances into the brain through tight junctions (TJs) and limited transcytosis, it simultaneously restricts the crossing of pharmaceuticals designed to act beyond this barrier *via* selective drug efflux transporters.^2^ The design of molecules that both cross the BBB and target disease mechanisms has proven difficult, especially considering the currently available models. Despite their widespread use, animal models do not fully recapitulate human BBB function and disease, which is one reason for the low translational success rate of drugs shown to be effective in animals.^3^ Alternatively, human-based *in vitro* models such as 2D cell monolayers or membrane-based co-cultures can be used. However, these often lack the necessary complexity and functionality of the brain vasculature and the surrounding cells and structures, collectively known as the neurovascular unit (NVU).

In recent years, there has been a substantial increase in publications describing *in vitro* BBB models using microfluidic devices: biocompatible, miniature engineered systems designed to mimic functional biological systems or processes.^4^ When combined with biomaterials such as hydrogels, these systems enable the culture of cells or tissue structures in a complex, physiologically relevant 3D environment. Moreover, cell culture medium can be automatically and dynamically supplied *via* automated pumps or by using gravity-driven flow.^5^ In the context of BBB modeling, combining three-dimensionality and flow can create an *in vivo*-like environment that respects vessel-like geometry and physiological shear stress, both of which are necessary for optimal BBB functioning.^6^ BBB-on-chip models can potentially predict if and how compounds cross the BBB with higher accuracy than in simpler systems.^7^ However, they often lack a direct connection to the brain parenchyma, which is also highly complex and affects BBB functioning, especially in the context of disease. Combining BBB-on-chip models with a complex, brain-like environment can substantially improve the relevance of these models for both disease modeling and drug testing.

Human brain organoids (hBOs) offer a brain-like microenvironment: complex, self-assembling *in vitro* tissue structures that resemble the developing human brain.^8^ In recent years, hBOs are increasingly used for a variety of research purposes, including the modeling of neurodegenerative diseases.^9^ However, hBOs inherently lack vasculature, which limits the study of important neurovascular processes and promotes the formation of a necrotic core due to insufficient oxygen and nutrient diffusion.^10^ Vascularized hBOs attempt to overcome these limitations. They can be generated by co-culturing hBOs with endothelial cells (EC) pre- or post-aggregation,^11–13^ or by fusion with blood vessel organoids.^14,15^ Moreover, hBOs can be cultured on microfluidic chips to facilitate vascularization by angiogenesis.^16–18^ However, vascularization of the hBO core still falls short,^10^ preventing the mitigation of the necrotic core. Furthermore, vascularized hBOs do not possess a functional or mature BBB,^10^ as newly sprouting vessels formed by angiogenesis exhibit higher transcytotic rates and are therefore more permeable compared to a fully developed BBB.^19,20^

Here, we describe the design and development of the CONNECT microfluidic chip, a human induced pluripotent stem cell (iPSC)-based co-culture system of sliced hBOs functionally linked to a biofabricated vessel representing the BBB. Using our system, we aimed to build a functional, controllable, and complex 3D *in vitro* model of the human NVU, without relying on angiogenesis. The CONNECT system has potential for advanced *in vitro* modeling of the human NVU in a healthy and diseased context, as well as testing if and how compounds cross the BBB and subsequently affect the brain environment *in vitro*.

## 2. Materials & methods

### iPSC culture for hBOs

We used two isogenic iPSC lines derived from the same donor (female, 60 years) using a different reprogramming method (Sendai virus or lentivirus for iPSC lines 1 and 2, respectively). In addition, we used iPSC lines derived from individual healthy control donors: line 3 (male, 61 years), line 4 (male, 62 years) and line 5 (male, 49 years). We also used an iPSC line derived from an Alzheimer′s disease patient with APOE4/4 genotype, CRISPR-Cas9-corrected to APOE3/3 (iPSC line 6; female, 72 years) that was previously described^21^ (ID: TCW1E33-1F1). iPSC lines, except for iPSC line 6, were obtained in-house with the written consent of donors and with approval of the Medical Ethical Committee of the University Medical Center Utrecht. iPSCs were generated as previously described.^22^ These lines were maintained feeder-free in Stemflex medium (Thermo Fisher, A3349401) on dishes coated with Geltrex (Thermo Fisher, A1413202). Media were refreshed 3-4 times weekly, and cells were split at 80% confluency using 0.5 mM EDTA (Thermo Fisher, 15575020) followed by culturing in Stemflex + 5 μM Y-27632 (Axon Medchem, 1683) for 24 h. iPSCs were regularly tested for mycoplasma with the MycoAlert kit (Lonza Bioscience, LT07-318). Genomic stability of iPSC lines was assessed by detecting recurrent genetic abnormalities using the iCS-digital PSC test, provided as a service by Stem Genomics. No chromosomal aberrations were detected in iPSC lines 1 and 3. Chromosomal aberrations were found in iPSC lines 4 and 5, with a trend (*p* < 0.05) towards gain in chromosome arms 1p and 19p (iPSC line 4), and statistically significant (*p* < 0.01) gains in 17p (iPSC line 4) and 12p (iPSC line 5). Genomic stability in iPSC lines 2 and 6 was not assessed.

### Unguided hBO differentiation

Unguided hBOs, also referred to as cerebral organoids or unguided neural organoids, were generated as previously described,^22^ based on the original protocol by Lancaster *et al.*^23^ with minor modifications. In short, 3.5 million iPSCs were cultured in one well of an Aggrewell™ 800 plate (StemCell Technologies, 27865) coated with anti-adherence solution (StemCell Technologies, 07010) in HES4+ medium from day 0 to 4. On day 2, embryoid bodies (EBs) were individually transferred to 96-well plates. On day 4, medium was replaced with HES0 medium, and on day 6, medium was replaced with neural induction medium (NIM) to induce neuroectodermal differentiation. On day 13, EBs were embedded in 100% Matrigel® (Corning, 356234). On the same day, the medium was replaced with B27-medium. From day 17 on, hBOs were kept in B27+ medium on an orbital shaker at 60 rpm. Medium was refreshed every 2–3 days. A list of all medium components is provided in Table S1.

### Cortical hBO differentiation

Cortical hBOs were generated as previously described^24^ with minor modifications. In short, a single-cell suspension of 9000 iPSCs in HES4+ medium was seeded in individual wells of an ultra-low attachment 96-well plate, allowing self-aggregation of EBs. At 2 DIV and 4 DIV, medium was replaced with fresh HES0 medium supplemented with 2.5 μM Dorsomorphin (R&D Systems, 3093) and 10 μM SB-431542 (Axon Medchem, AXON 1661). At 6 DIV, hBOs were cultured in neural medium supplemented with 20 ng mL^−1^ EGF (R&D Systems, #236-EG) and 20 ng mL^−1^ bFGF (PeproTech, 100-18B). At 25 DIV, hBOs were cultured in neural medium supplemented with 20 ng mL^−1^ BDNF (STEMCELL Technologies, 78005.1) and 20 ng mL^−1^ NT-3 (PreproTech, 450-03B). Thereafter, hBOs were allowed to further mature in the 96-well plate in plain neural medium before initiating slicing. Medium was replenished every 2–3 days. A list of all medium components is provided in Table S1.

### Live slicing of hBOs

Unguided and cortical hBOs were sliced into live sections as previously described.^25,26^ In short, hBOs were washed in ice-cold 1× HBSS (Gibco, 14065049) and embedded in 3% low-melting agarose (Fisher Bioreagents, BP165-25) solution kept at 50 °C in 1× HBSS before allowing to polymerize on an ice block for 15–20 min. An agarose block containing multiple hBOs was glued to the holder of a vibratome. hBOs were sliced while suspended in ice-cold 1× HBSS on the vibratome into 200–250 μm thick sections (speed 0.12 mm s^−1^, frequency 45 Hz) and allowed to recover in the incubator for 1–3 h in recovery medium. After recovery, hBO slices were immediately placed in the chip, on a membrane insert, or cultured free-floating in air–liquid interface cerebral/cortical organoid (ALICO) medium until further use. A list of all medium components is provided in Table S1.

### hCMEC/D3 culture

A human cerebral microvascular endothelial cell (hCMEC/D3) line^27^ was provided by P.O. Couraud (Institut Cochin, Paris, France). As previously described,^28^ cells were cultured on collagen I (Sigma, C9791) coated plates in the EGM®-2 MV microvascular endothelial cell growth medium-2 BulletKit® (Lonza, CC-3202) (from now on referred to as EGM-2 medium). Medium was replenished every 2–3 days, and cells were passaged at 80–100% confluence using Trypsin–EDTA (Gibco, 15400054). Cells were used for experiments up to 35 passages.

### Membrane insert co-culture of cortical hBO slices and hCMEC/D3 cells

The bottom of a 24-well, polycarbonate membrane Transwell® insert with 0.4 μm pore size (Corning, CLS3413) was treated with 0.1 mg mL^−1^ poly-d-lysine (Thermo Scientific, A3890401) in the incubator overnight, washed twice with sterile H_2_O, and dried at room temperature for 30 min. The bottom of the same insert was then coated with 50 μg mL^−1^ mouse laminin (Sigma-Aldrich, L2020) in 1× Dulbecco's PBS (DPBS) with Mg^2+^ and Ca^2+^ (Gibco, 14040141) at 4 °C overnight. Cortical hBO slices (iPSC line 1, 107 DIV), previously kept in recovery medium on an orbital shaker (60 rpm) overnight, were placed on the bottom of the membrane insert using a wide-bore pipette tip in a 10 μL droplet of ALICO medium. To allow for slice attachment, the insert was kept upside-down in a well of a 12-well plate with an elevated lid in the incubator for 1 h. To prevent the slice from drying out, a 10 μL droplet of ALICO medium was added every 10 minutes. After successful attachment, the insert was flipped into the correct position in a 24-well plate, submerged in 500 μL of ALICO medium, and placed in the incubator. Medium changes were performed three times per week. 14 days later, at 121 DIV, the opposite (top) side of the insert was coated with 50 μg mL^−1^ mouse laminin in the incubator for 4 h. Laminin solution was removed and replaced with 50 000 hCMEC/D3 cells in EGM-2 medium, after which cells were allowed to attach. Both media (ALICO and EGM-2) were replenished every 2–3 days. At 128 DIV, after 7 days of co-culture, hCMEC/D3 cells and hBO slice on the insert were fixed in 4% paraformaldehyde (PFA) in 1× PBS at 4 °C overnight, and washed three times with 1× PBS the next day. The membrane of the insert, containing the attached hCMEC/D3 cells and hBO slice, was cut out using a scalpel and stored in 1× PBS at 4 °C until staining. A list of all medium components is provided in Table S1.

### iPSC culture for endothelial cells (hiECs) and brain pericytes (hiBPCs)

iPSC line 7 is from an Alzheimer's disease patient with APOE4/4 genotype (male, 80 years) that was previously described and obtained from the authors^21^ (ID: TCW2E44-4B1). iPSC line 8 was generated as previously described.^29^ iPSCs were maintained feeder-free in mTeSR Plus medium (STEMCELL Technologies, 00-0276) on dishes coated with 0.5 μg cm^−2^ vitronectin (Thermo Fisher, A14700). Media were refreshed 3 times per week, and cells were passaged in clumps (passage ratio of 1 : 20–1 : 30) at 80% confluency using Versene EDTA solution (Gibco, 15040066).

### Differentiation of hiECs

iPSC lines 7 and 8 were differentiated into naïve endothelial cells as described previously^29^ using an adaptation of previously published protocols.^30,31^ Briefly, iPSCs with a confluency of 70–80% were detached using Versene and pipetted up-and-down to obtain clumps with a size of 0.5–1 mm. iPSC clumps were seeded onto vitronectin-coated plates or flasks at a passage ratio of 1 : 20–1 : 30, depending on the confluency and growth speed of the iPSC line. iPSCs were cultured for 24 h in mTeSR Plus. At day 0 of differentiation, mesoderm induction was started by changing the medium to B(P)EL medium (Orlova *et al.*, 2014)^30^ with 8 μM CHIR 99021 (CHIR; Tocris, 4423). At day 3 of differentiation, vascular specification was started by changing the medium to B(P)EL with 50 ng mL^−1^ vascular endothelial growth factor (VEGF; Biolegend, 583206) and 10 μM SB431542 (Selleck Biotechnology, S1067). Medium was refreshed on days 6 and 8. At day 10 of differentiation, cells were detached using Accutase (STEMCELL Technologies, 07922), centrifuged at 300*g* for 5 min, counted, and magnetically-activated cell sorted for CD31 using the CD31 MicroBead Kit (Miltenyi Biotec, 130-091-935), the QuadroMACS™ Separator (Miltenyi Biotec, 130-091-051), and the LS columns (Miltenyi Biotec, 130-042-401), following the manufacturer's instructions. CD31^+^ hiECs were counted and frozen in CryoStor CS10 (STEMCELL Technologies, 07930), or seeded at a density of 1.5 × 10^4^ cells per cm^2^ onto plates or flasks coated with 10 μg mL^−1^ human placenta-derived collagen IV (Sigma-Aldrich, C5533) in endothelial serum-reduced medium ++ (ECSR++), comprising of human endothelial serum-free medium (Gibco, 11111044), 1% human serum derived from platelet-poor plasma (Sigma-Aldrich, P2918), 30 ng mL^−1^ VEGF, and 20 ng mL^−1^ basic fibroblast growth factor (bFGF; Peprotech, 100-18B). hiECs were cultured in ECSR++ and passaged up to 3 times at 1 : 4 ratio when 100% confluency was reached.

### Differentiation of hiBPCs

iPSC line 8 was differentiated into neural crest (NC)-derived hiBPCs as described previously^29^ using an adaptation of previously published protocols.^32,33^ Briefly, iPSCs were passaged as single cells and seeded at a density of 2 × 10^5^ cells per cm^2^ onto vitronectin-coated plates. Cells were cultured in NC induction medium, consisting of DMEM/F12 GlutaMAX™ (Gibco, 10565018), 1× B-27 (Gibco, 17504044), 0.5% BSA, and 3 μM CHIR for 5 days, with daily media refreshments. 10 μM ROCK-inhibitor (Y-27632; Selleck Biotechnology, S1049) was added on the first day. The resulting NC cells were detached with Accutase and frozen in CryoStor CS10 or directly seeded onto 0.1% gelatin-coated plates at a density of 2.5 × 10^4^ cells per cm^2^. NC cells were cultured in pericyte medium (PM; ScienCell, 1201) for 5 days for BPC specification, with daily refreshments. Immunocytochemistry and mRNA evaluation of PDGFRβ, NG2, CD13, CD146, FOXF2, and FOXC1 confirmed their pericyte identity. hiBPCs were used between passages 3 and 6.

### Membrane insert co-culture of cortical hBO slice and hiECs

The bottom of a 96-well polycarbonate membrane Transwell® insert with 0.4 μm pore size (Corning, CLS3391) was treated with PDL and coated with mouse laminin as described above. Cortical hBO slices (iPSC line 6, 136 DIV) were attached to the bottom of the insert as described above. For hiEC seeding, the opposite (top) side of the insert was coated with 100 μg mL^−1^ human collagen IV (Sigma-Aldrich, C5533) in sterile water in the incubator for 60 min. 10 000 hiECs in ECSR++ medium were plated and allowed to attach as described above. Co-culture was maintained for 7 days.

### Staining and tissue clearing of membrane insert co-culture of cortical hBO slice and hCMEC/D3 cells

The membrane insert containing the hCMEC/D3 and hBO slice co-culture was stained and cleared for 3D imaging using an adaptation of an existing protocol.^25,34^ A list of all solutions and their components is provided in Table S2. A list of all used antibodies can be found in Tables S3 and S4. The insert was blocked against non-specific binding in washing buffer 1 (WB1) on a shaker for 4 h at room temperature. WB1 was removed and replaced with a primary antibody mix in washing buffer 2 (WB2) containing 50% of the desired final primary antibody concentration and incubated on a shaker at room temperature overnight. The next day, primary antibodies were replenished to obtain their final concentrations and incubated on a shaker at room temperature overnight. The following day, the primary antibody mix was removed, and the insert was washed with WB2 on a shaker at room temperature for 1 h. This step was repeated 2 additional times before WB2 was replaced with a secondary antibody mix in fresh WB2 containing 50% of the desired final secondary antibody concentration and incubated on a shaker at room temperature overnight. The next day, secondary antibodies were replenished to obtain their final concentrations and incubated on a shaker at room temperature overnight. The following day, the secondary antibody mix was removed, and the insert was washed with WB2 on a shaker at room temperature for 1 h. This step was repeated 2 additional times. For tissue clearing, WB2 was replaced with 33% FUnGI clearing solution in WB2 and incubated on a shaker at room temperature for 1 h. Then, 33% FUnGI was replaced with 66% FUnGI in WB2 and incubated on a shaker at room temperature for 1 h. Lastly, 66% FUnGI was replaced with 100% FUnGI and incubated on a shaker at 4 °C for ∼3 days. The stained and cleared insert was mounted between two glass coverslips separated by iSpacers 0.25mm (SUNJin Lab, IS213) in 100% FUnGI solution.

### Fabrication of microfluidic chips

Devices were fabricated using BioMed Amber Resin (Formlabs) and a Form 3B+ stereolithography printer. CAD designs were exported as STL files and printed at a layer thickness of 50 μm. Upon completion, printed parts were washed in 99% isopropanol using the form wash system for 15 min to remove residual uncured resin, followed by immersion in fresh isopropanol for an additional 5 min. Parts were then removed from the solvent and allowed to air dry at room temperature for 30 min. Dried prints were post-cured in the form cure chamber at 60 °C for 15 min in accordance with manufacturer specifications. After curing, printed components were permanently bonded to glass coverslips using Loctite M-31CL epoxy, and assemblies were left to fully cure before downstream use.

### Quality control of fabricated chips

Structural quality of the fabricated devices was assessed by brightfield microscopy. Dimensional variance across replicated devices was consistently below 10 μm (<5%), and microstructural features generated from the stereolithography masters were retained after multiple replication cycles. Bond integrity was evaluated under conditions mimicking cell culture. Devices filled with sterile water or phosphate-buffered saline were incubated overnight at 37 °C. Assemblies that remained fully sealed after incubation were deemed suitable for downstream long-term cell culture experiments.

### Device sterilisation

Assembled devices were sterilised prior to use by adding 70% ethanol to all inlets and outlets (1 h + overnight drying for any cultures involving hBOs, rapid flushing for other cultures). Devices were then exposed to ultraviolet light for 15–30 min in a biosafety cabinet. Following sterilisation, devices were rinsed three times with sterile 1× phosphate-buffered saline (PBS) (137 mM NaCl, 2.7 mM KCl, 10.1 mM Na_2_HPO_4_·2H_2_O, and 1.76 mM KH_2_PO_4_ in demineralized water, pH 7.4) to remove ethanol residues.

### Capillary pressure barrier loading

The chip glass surface was treated with 0.1 mg mL^−1^ poly-d-lysine (Thermo Scientific, A3890401) in the incubator overnight, washed twice with sterile water, and dried at room temperature for 30 min in a biosafety cabinet. 2 μL of 4 mg mL^−1^ rat tail collagen I hydrogel (Advanced Biomatrix, 5153) were carefully dispensed along the capillary pressure barrier (CPB) from the hBO chamber side and incubated at 37 °C for 15 minutes in a humidified environment. After hydrogel polymerization, 3 μL of 100 μg mL^−1^ collagen IV were added to the microfluidic channel from the BBB channel inlet. 20 μL of PBS were added to the hBO chamber to avoid gel shrinkage. Chips were incubated at 37 °C for 1 h.

### Microfluidic flow simulation

A lumped-parameter analytical model was used to estimate gravity-driven perfusion, flow decay, and wall shear stress in a rocker-operated microfluidic chip, based on previous reports.^35,36^ The modeled geometry comprised two square reservoirs with a cross-sectional area of 6 × 6 mm connected by a single straight channel with dimensions 0.5 × 0.5 × 9.8 mm (width × height × length). The working fluid was assumed to be culture medium at 37 °C, represented as an incompressible Newtonian liquid with density 1000 kg m^−3^ and dynamic viscosity 0.69 mPa s.

The rocker protocol was modeled as bidirectional interval tilting at ±14° with a reversal every 4 min, and the flow profile was simulated over a 12 min window, corresponding to three consecutive rocking intervals. At the start of each interval, the hydrostatic head difference between reservoirs was calculated from the vertical offset imposed by tilt, Δ*h* = *L* sin(*θ*)where *L* is the reservoir-to-reservoir distance and *θ* is the tilt angle. The corresponding pressure drop was obtained from Δ*P* = *ρg*Δ*h* where *ρ* is fluid density and *g* is gravitational acceleration.

Channel hydraulic resistance was estimated using the standard rectangular microchannel expression
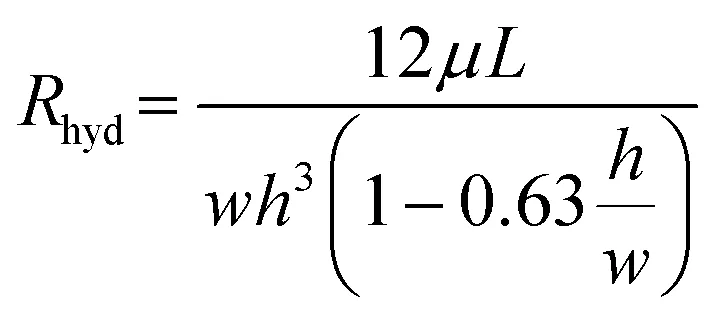
where *μ* is dynamic viscosity and *L*, *w* and *h* are channel length, width and height, respectively. Volumetric flow rate was then obtained from the hydraulic analogue of Ohm's law,
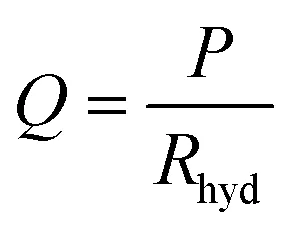
For time-dependent simulations, the reservoir system was treated as a head-equalization problem, producing an exponential decay in flow within each rocking interval. The characteristic decay time was calculated as
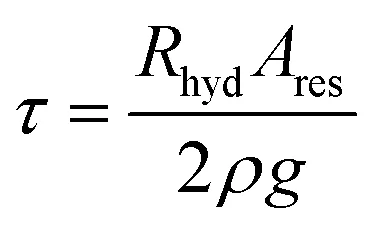
where *A*_res_ is the reservoir cross-sectional area.

Wall shear stress was estimated using the parallel-plate approximation for rectangular microchannels,
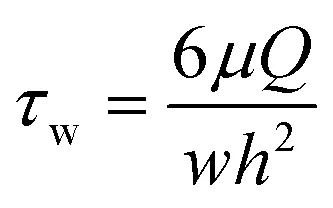
which is commonly used for microfluidic shear calculations when cells are cultured onto the channel wall. Flow rate and wall shear stress were computed at 0.25 s time steps for the full 12 min simulation. At each 4 min reversal, the model reset the hydrostatic driving head to the same magnitude with opposite direction, generating a repeating sequence of transient pulses.

The model assumes laminar flow, rigid channel walls, negligible entrance effects, negligible capillary effects, and no evaporation or density mismatch between the two reservoirs. Mixing within each reservoir was assumed to be instantaneous such that each reservoir could be represented by a uniform liquid column with a constant cross-sectional area. The resulting output variables were volumetric flow rate and wall shear stress as functions of time, reported in nL s^−1^ and dyn cm^−2^ (= 100 mPa), respectively.

### hiEC on-chip culture

100% confluent hiECs were detached using Accutase, counted, and resuspended in ECSR++ at a concentration of 10 × 10^6^ cells per mL. BBB channel coating solution was carefully aspirated with a 20 μL pipette. 3 μL of hiEC suspension was added to the BBB channel inlet. Chips were incubated at 37 °C for 2 h at a 45° angle towards the CPB to promote adhesion of hiECs to the gel. PBS in the hBO chamber was replaced with 400 μL endothelial serum-free medium (SFM; Gibco, 11111044). 300 μL of ECSR++ was added to the BBB channel inlet and outlet and chips were placed on OrganoFlow rocker (Mimetas B.V., MI-OFPR) set at 14 degrees with 4-minute intervals. The medium was refreshed every other day. After 7 days of culture, chips were fixed by aspirating media, adding 4% PFA to the BBB channel inlet (50 μL), outlet (100 μL), and organoid chamber (150 μL), and incubating at room temperature for 15 min.

### BBB-on-chip culture

BBB-on-chip was formed by co-culturing hiECs and hiBPCs in CONNECT chips and adding CHIR at day 5 to generate hiBMECs.^29^ hiBPCs were detached using Accutase, counted, and the required amount was centrifuged at 300 × *g* for 5 min. The supernatant was aspirated, the cell pellet was resuspended in collagen I hydrogel at a concentration of 2 × 10^6^ cells per mL, and 2 μL of the suspension were carefully dispensed along the CPB. Chips were placed in the incubator for 15 min to allow gel polymerization. hiECs were seeded as described above, with PM added to the organoid chamber instead of endothelial SFM. Medium was refreshed every other day. At day 5, CHIR (4 μM) was added to the BBB channel. After 7 days of co-culture, chips were fixed by aspirating media, adding 4% PFA to the inlet (50 μL), outlet (100 μL), and organoid chamber (150 μL), and incubating at room temperature for 15 min.

### Whole unguided hBO on-chip culture

CPB was loaded with 100% Matrigel (Corning, 356234), which was allowed to polymerize in an incubator for 20 min. A single, whole, unguided hBO at 200+ DIV (iPSC line 2) was placed in the organoid chamber with B27+ medium. A list of all medium components is provided in Table S1.

### Viral transduction of sliced cortical hBO (on-chip) and whole cortical hBO (off-chip)

To visualize GFAP-expressing cells (*i.e.*, astrocytes) in hBOs, we transduced hBOs or hBO slices with an AAV expressing eGFP under the human GFAP promoter gfa2. pAAV.GFAP.eGFP.WPRE.hGH (lot number v127507) was a gift from James M. Wilson (Addgene viral prep # 105549-AAV5; http://n2t.net/addgene:105549; RRID: Addgene_105549). We transduced hBOs or hBO slices with 3.125 μL (titer 1.6 × 10^13^ GC mL^−1^) virus in 500 μL of ALICO medium in the incubator for 48 h. A list of all medium components is provided in Table S1.

### Sliced cortical hBO on-chip culture

The chip glass surface was treated with 0.1 mg mL^−1^ poly-d-lysine in the incubator overnight, washed twice with sterile water, and dried at room temperature for 30 min in a biosafety cabinet. CPB was loaded with rat tail collagen I hydrogel. hBOs of iPSC lines 1 and 3 (239 DIV) were sliced on the day of chip attachment, unless otherwise indicated. The chamber bottom was coated for 1 h at 37 °C with a 1 : 1 mixture of 50 μg mL^−1^ mouse laminin (Sigma-Aldrich, L2020) in 1× DPBS with Mg^2+^ and Ca^2+^ (Gibco, 14040141) and 100 μg mL^−1^ human collagen IV (Sigma-Aldrich, C5533) in sterile water. The coating solution was removed, and the hBO chamber bottom was washed twice with 1× PBS. hBO slices were added to the chamber with a cut p200 tip, the slice was positioned as close as possible to the CPB, and the medium was changed from recovery medium to ALICO medium (500 μL). Two days after the start of culture, the medium was refreshed. Cultures were fixed with 4% PFA after 5 days of culture. A list of all medium components can be found in Table S1.

### NVU-on-chip culture

Viral transduction of hBOs was done 48 hours before slicing. CONNECT chips were coated as described in the section above. hiECs and hiBPCs were added to the chips as described in the BBB-on-chip culture section. hBOs were sliced and added to the chips as described in the section above. Media (ALICO and ECSR++) were refreshed two days after the start of co-culture and fixed after 5 days of co-culture.

### On-chip immunocytochemistry

Chips were carefully rinsed with PBS three times, and 4% PFA was added to the inlet, outlet, and organoid chamber for 15 min. Cells were permeabilized for 10 min using 0.3% Triton X-100 (Sigma-Aldrich, X100) in PBS. Cells were then blocked with 10% normal donkey serum (Jackson ImmunoResearch, 017-000-121) in 0.05% Tween-20 (Sigma-Aldrich, P9416) in PBS (from now on referred to as PBSDK) for 30 min. Primary antibodies were diluted in 1% PBSDK, and cells were incubated in the antibody solutions at 4 °C overnight. After three PBS washes, cells were incubated with secondary antibodies and DAPI in 1% PBSDK at room temperature for 4 h. Cells were then washed with PBS three times. A detailed list of antibodies is shown in Tables S3 and S4.

### Microscopy

Images of live hBO slices, Transwell® cultures, and chip co-cultures (brightfield, GFP, Texas Red) were captured regularly using an EVOS M5000 microscope (ThermoFisher, AMF5000). 3D fluorescent stainings of cleared tissues were imaged on the LSM880 (Zeiss). CONNECT chip live imaging was done on the THUNDER Imager 3D Live Cell (Leica). On-chip immunocytochemistry was performed using the AX R confocal microscope (Nikon). Images were edited for clarity by adjusting brightness (min/max) and contrast in Fiji. Some images were merged using the grid/collection stitching plugin (default settings). For GFAP-GFP quantification, the mean fluorescence intensity (MFI) of the green channel was quantified at all timepoints, and the ratio between final and initial timepoints was calculated by dividing the final MFI by the initial MFI. 3D reconstruction was done using Imaris 9.5 software (Bitplane, Oxford Instruments).

### Permeability assay on-chip

Sodium fluorescein (10 μm; NaFl, Sigma-Aldrich) was diluted in ECSR++ and added to the BBB channel inlet and outlet. Chips were incubated for 1 h at 37 °C, and a calibration curve of serial dilutions of NaFl was prepared in a black-walled 96-well plate. Samples from the hBO compartment were collected, and the fluorescence was measured using a Synergy HTX Multimode Reader (Biotek), with excitation/emission wavelengths of 485/530 nm. Apparent permeability coefficients (*P*_app_) were calculated using the following equation: *P*_app_ = (*C*_b_ × *V*_b_)/(*C*_a_ × *A* × Δ*t*), where *C*_b_ is the concentration measured in the hBO compartment at 1 h, *V*_b_ is the volume of medium in the hBO compartment (0.4 mL), *C*_a_ is the concentration in the BBB channel at 0 h (10 μm), *A* is the surface area available for permeability (0.45 cm^2^) and Δ*t* is the duration of the experiment (3600 s).

### P-glycoprotein activity assay on-chip

The P-gp activity was evaluated by assessing the permeability to rhodamine 123 (Rho_123_), a fluorescent P-gp substrate, with or without the presence of verapamil, a P-gp blocker. The respective groups were pre-incubated with 25 μm verapamil (Sigma-Aldrich) diluted in ECSR++ and added to the BBB channel inlet and outlet 1 h prior to Rho_123_ treatment. After 1 h, 10 μm Rho_123_ (Sigma-Aldrich) diluted in ECSR++ was added to the BBB channel inlet and outlet, and chips were incubated for 1 h at 37 °C. Samples from the hBO compartment were collected in a black-walled 96-well plate, and the fluorescence was measured using a Synergy HTX Multimode Reader, with excitation/emission wavelengths of 485/530 nm. Concentrations of Rho_123_ were determined by standard calibration curves. *P*_app_ coefficients were calculated as described above. P-gp activity coefficient was calculated using the equation (*P*^VP^_app_/*P*^UNT^_app_) − 1, where *P*^VP^_app_ is the Rho_123_*P*_app_ on VP-treated cells, and *P*^UNT^_app_ is the Rho_123_*P*_app_ on untreated counterparts.

### Cell alignment and junction quantification

10 fluidic units were seeded with hiECs as described above and cultured for 7 days under flow (bidirectional flow, 14 degrees tilt with 4-minute intervals, as described above) or static conditions (5 fluidic units per condition). On-chip hiECs were fixed and stained, as described above, for VE-cadherin and DAPI. Image analysis was performed using Polarity-JaM;^37^ instance segmentation was generated with the integrated CellposeSegmenter using the DAPI (nuclei) and VE-cadherin (junction) on Cellpose3 model.^38^ Objects smaller than 50 px^2^ or touching the image border were excluded from the analysis. VE-cadherin^+^ pixels within each interface ROI were identified by Otsu thresholding applied locally, yielding the junction protein area, while the interface area was defined as the area of the dilated outline. From these measurements we computed interface occupancy (junction protein area/interface area), intensity per interface area (mean VE-cadherin intensity across the interface ROI after background subtraction using the image histogram mode), and cluster density (mean VE-cadherin intensity within the thresholded junction protein area). Cell morphology features were extracted from segmentation masks and included cell area, major and minor ellipse axes (from which length-to-width ratio was derived), circularity (4π*A*/*P*^2^) and shape index (*P*/√*A*), and orientation of the cell major axis. Local junction orientation fields were computed from the VE-cadherin channel using a structure-tensor method. Angular statistics, circular histograms and polarity indices (normalized resultant vector length, 0–1) were produced with Polarity-JaM circular statistics routines and the web app (https://polarityjam.com), treating axial data as described in the Polarity-JaM documentation. All analyses were run in batch *via* the Polarity-JaM CLI, producing per-image CSV files and QC images.

### Statistics

Data were statistically analyzed using GraphPad Prism v10 (GraphPad Software). Normal distribution was determined using Shapiro–Wilk test. For datasets with 2 groups, two-tailed paired or Welsh's *t*-test was used. For datasets with 3 groups, one-way ANOVA was used.

## 3. Results

### hBO slice-derived astrocytes sprout towards and physically contact ECs in a membrane co-culture

**Fig. 1:**
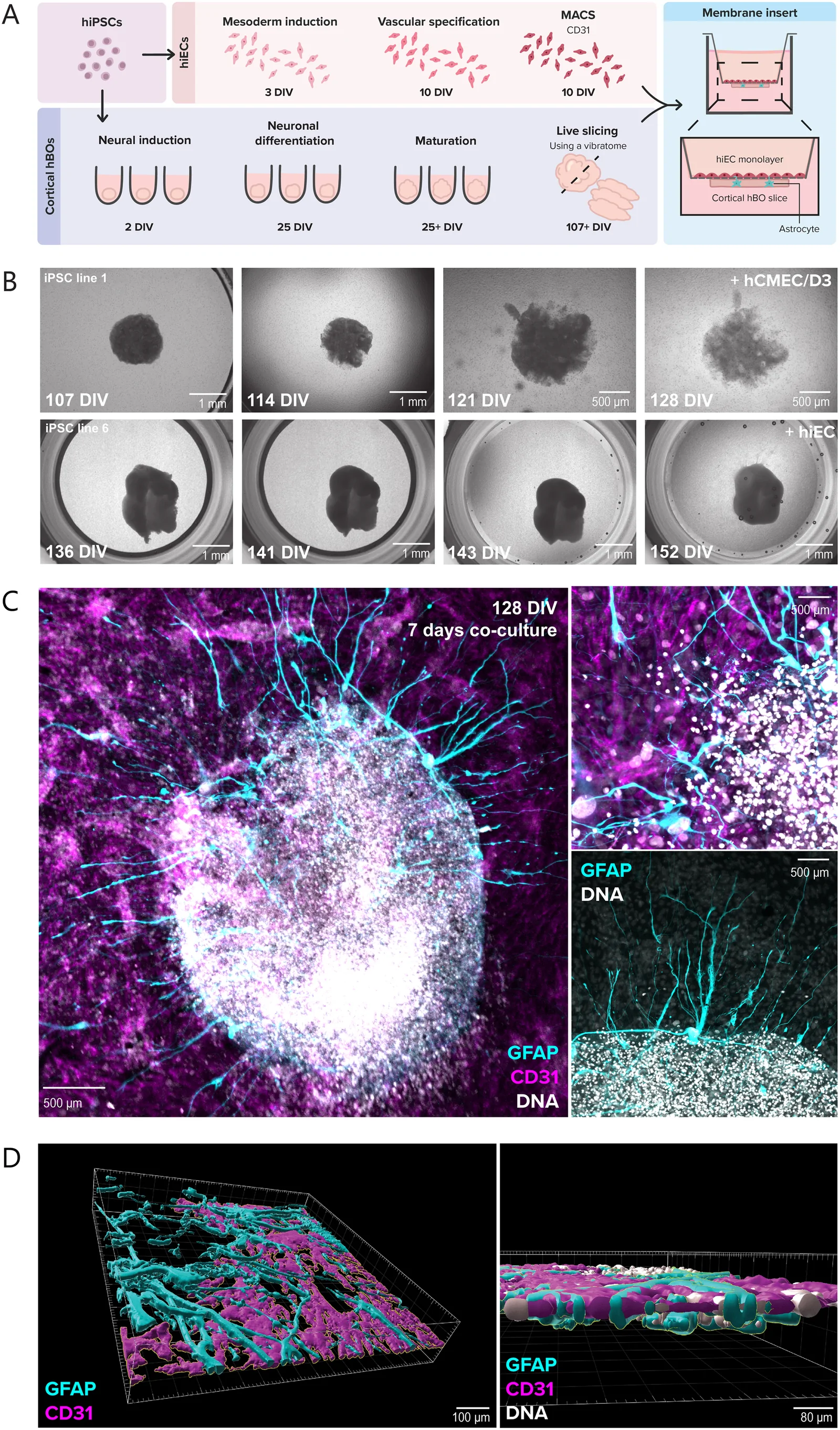
hBO-derived astrocytes sprout toward and physically interact with ECs on a membrane insert. A. Schematic overview of a co-culture of ECs (top) and a cortical hBO slice (bottom) on a membrane insert. B. Brightfield images of cortical hBO slices of iPSC lines 1 (left, 107-128 DIV) and 6 (right, 136-152 DIV) on membrane inserts over time, before and after co-culturing with ECs: hCMEC/D3s (added at 121 DIV) and hiECs (added at 143 DIV; iPSC line 7). C. Confocal images of the membrane insert co-culture system (hCMEC/D3 and iPSC line 1 cortical hBO at 128 DIV) 7 days after start of co-culture, cleared and stained for GFAP (cyan) and CD31 (magenta). Nuclei were counterstained with Hoechst (white). D. Three-dimensional reconstruction (left panel: bottom-up; right panel: side view) of the staining depicted in C.

Previous studies have shown that 2D astrocytes can sprout their processes towards ECs through a porous membrane.^39^ To evaluate if astrocytes present in hBOs could replicate this response, we created a co-culture of monolayered ECs (hCMEC/D3 or hiECs) (top) and a live hBO slice (bottom) on a membrane insert (Fig. 1A). We used a small pore size (0.4 μm) to prevent EC monolayer disruption while enabling astrocyte processes to sprout toward and contact the ECs. Slices of freshly vibratome-sectioned hBOs adhered and remained attached to the bottom of a PDL-laminin-coated membrane insert (Fig. 1B). 14 days later, we added ECs on the opposite side of the insert and co-cultured them for 7 days. Notably, we observed sprouting and migration of hBO slice-derived cells, regardless of hBO slice age or EC type used (Fig. 1B).

To better visualize this and confirm the identity of migrating/sprouting cells, we fixed, cleared, and stained the membrane after 7 days of co-culture. Our staining confirmed the presence of a CD31^+^ EC monolayer, and we observed that part of the cells sprouting from the hBO slice were positive for astrocyte marker GFAP (Fig. 1C). Moreover, astrocyte processes sprouted outward through the membrane and physically contacted ECs (Fig. 1D). Hereby, we obtained proof-of-principle that hBO-derived astrocytes can sprout from an hBO and physically contact ECs.

### Design of a membrane-free microfluidic chip enabling hydrogel-based compartmentalization of an open-top chamber and a vessel-like channel

**Fig. 2:**
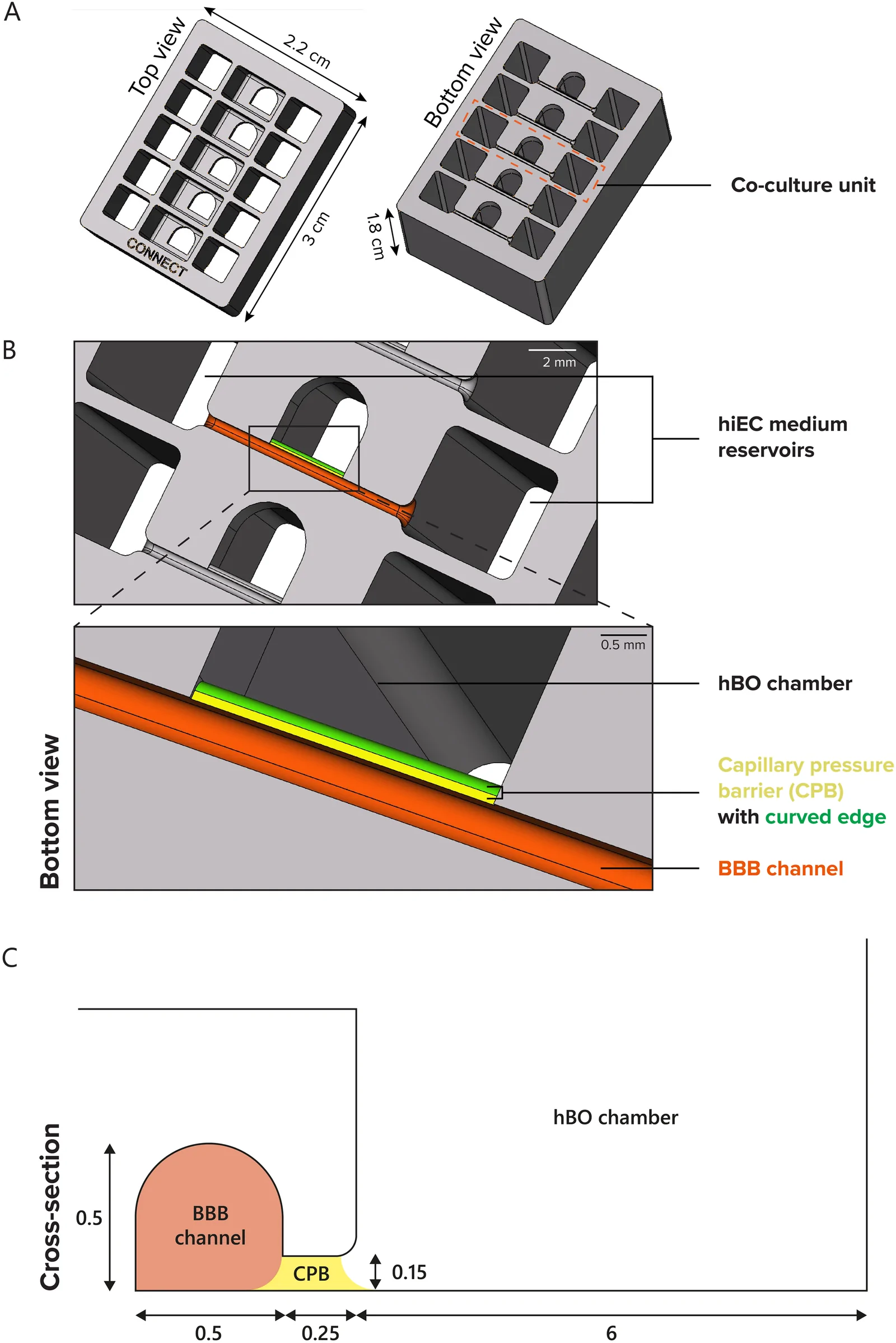
Design of the CONNECT microfluidic chip. A. Top and bottom view of the CONNECT chip (2.2 × 3.0 × 1.8 cm *L* × *W* × *H*) containing five co-culture units (red dotted line). B. Bottom view of a co-culture unit within the CONNECT chip, highlighted. One co-culture unit is composed of a three-rounded-corner quadrilateral-shaped BBB channel (red; 9.8 × 0.5 × 0.5 mm *L* × *W* × *H*), capillary pressure barrier (CPB; yellow; 3 × 0.25 × 0.15 mm *L* × *W* × *H*) including curved edge (green), and an open-top hBO chamber (3.5 × 1.5 mm diameter × *H*). Medium for hiECs can be supplied using bi-directional, gravity-driven flow through the BBB channel *via* two medium chambers on either side of the channel. C. Cross-section (side view) visual representation of the CONNECT chip, including dimensions (mm). The single-corner CPB allows the formation of a meniscus at the BBB channel interface, resulting in a luminal-shaped BBB channel with one single sharp edge (bottom left corner).

After obtaining proof-of-principle that astrocytes from an hBO slice can sprout towards and contact ECs, we designed a microfluidic chip that enables a more complex and structurally relevant co-culture between the BBB and hBOs — the CONNECT microfluidic chip (Fig. S1A). For the chip material, we chose BioMed Amber Resin, a robust, semi-transparent, and biocompatible material applicable for drug testing due to low water absorption. The CONNECT microfluidic chip has a glass bottom for inverted microscopy and an open-top design for simplified cell culture medium changes (Fig. 2A). One chip contains five co-culture units. Each co-culture unit contains a BBB channel with vessel-like geometry, which is connected to the hiEC medium reservoirs (Fig. 2B). Adjacent to the BBB channel is the hBO chamber for attachment of an hBO (slice) on the glass bottom. The compartments are separated by a capillary pressure barrier (CPB). The CPB has a curved edge and is 150 μm high, meaning it can be loaded with a very low volume of hydrogel (3 μL) that remains in place due to the pinning effect generated by the CPB geometry (Fig. 2B–D). The curved edge allows for the formation of a meniscus along the *YZ* axis, resulting in a luminal-shaped BBB channel (Fig. S1B). Cells can be seeded directly in or on this hydrogel, eliminating the need for a porous membrane while providing a substrate for the formation of a monolayer of hiECs and allowing for direct physical contact between cells in the BBB channel and hBO chamber. When placed on a rocker device, the chip design allows pump- and tubing-free, gravity-driven, bi-directional flow of cell culture medium through the BBB channel, between inlet and outlet reservoirs. The peak flow rate in the BBB channel is 8.64 nL s^−1^, with a decay time constant of 4.94 s, resulting in a peak wall shear stress of 2.86 dyn cm^−2^ (Fig. S1C).

### Optimization of on-chip hBO culturing enables sprouting and migration of hBO slice-derived astrocytes

Astrocytes start to emerge in unguided hBOs at around 100+ DIV.^40^ Due to their size at this timepoint (∼3 mm diameter), hBOs require constant orbital shaking to limit core necrosis. To examine whether hBOs could be cultured long enough to obtain astrocytes in our static system whilst remaining viable, we initiated on-chip culture of a whole, unguided hBO at 13 DIV (Fig. S2A and B). At 20 DIV, we detected cells migrating from the hBO into the Matrigel, as commonly observed in regular hBO cultures.^23^ A population of cells was seen migrating towards the BBB channel despite the absence of cells or medium flow in this channel (Fig. S2B and C). Moreover, the on-chip hBO experienced a delay in growth when compared to hBOs in suspension culture (Fig. S2D). After 43 DIV, the on-chip hBO stopped showing signs of viability, such as growth and cell migration (data not shown).

Seeing that the static culture of early-stage hBOs limits growth and cell viability, we next attempted the on-chip culture of a more mature, astrocyte-containing whole hBO at 200+ DIV that had previously been cultured in regular, orbital shaking conditions. However, we noticed only a small cluster of hBO-derived cells sprouting into the hydrogel-laden CPB (Fig. S2E). Considering that sliced hBO cultures earlier showed a substantial astrocyte sprouting capacity (Fig. 1) and have better distributed accessibility to oxygen and nutrients compared to whole hBOs (Giandomenico *et al.*, 2019),^26^ we used hBO slices for on-chip culturing moving forward.

**Fig. 3:**
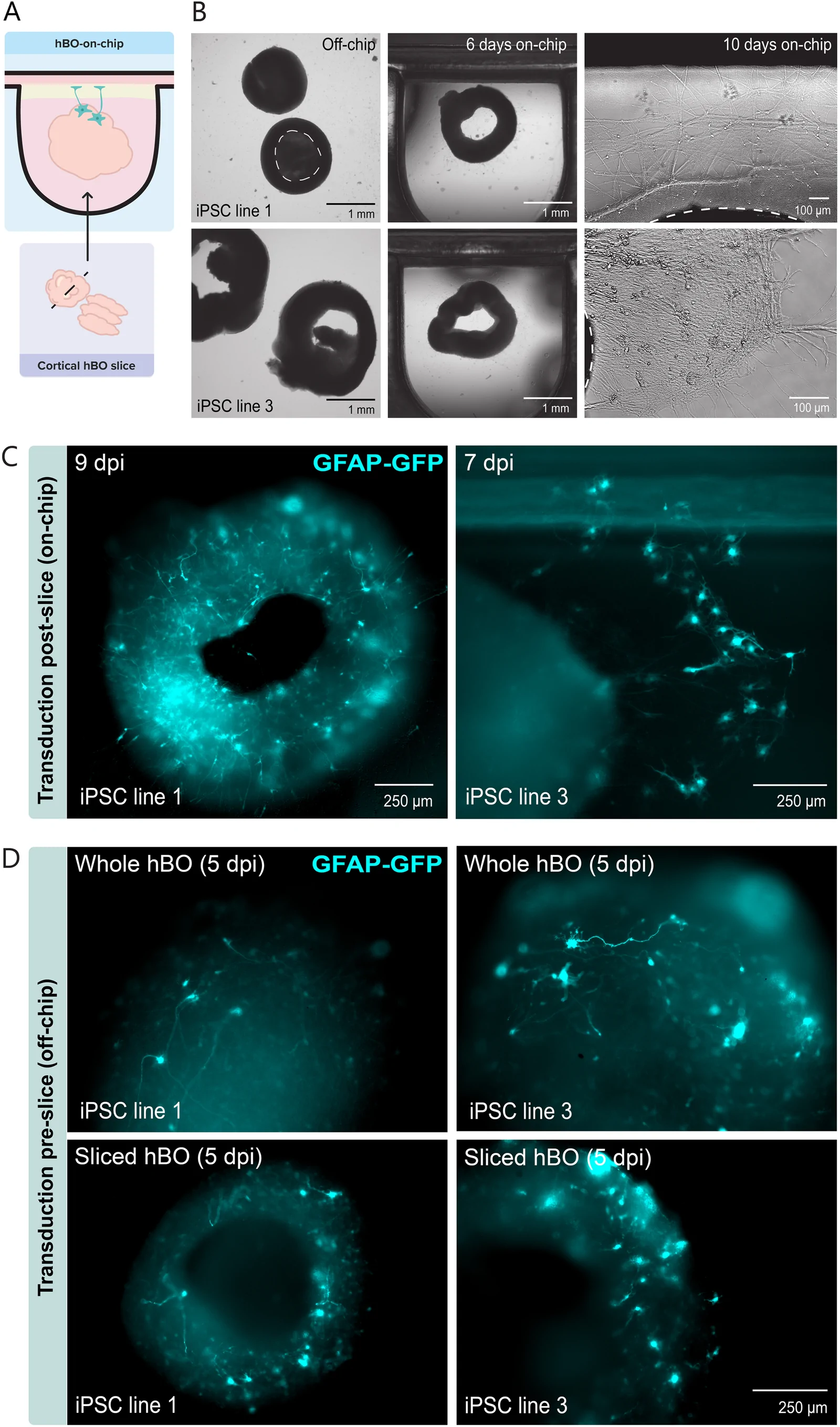
Attachment of sliced cortical hBOs on the CONNECT chip and visualization of hBO-derived astrocytes. A. Schematic illustration of the CONNECT cortical hBO-on-chip. B. Brightfield images of cortical hBO slices (150-171 DIV, iPSC lines 1 and 3) with a necrotic core (indicated by white dotted line) outside of the CONNECT chip (left panels) or attached on-chip (middle and right panels) on collagen IV/laminin (1 : 1) coating. C. Epifluorescent images of on-chip cortical hBO slices at 160-183 DIV (7–9 days post-infection; dpi) transduced with pAAV.GFAP.eGFP.WPRE.hGH, showing GFAP-GFP^+^ astrocytes (cyan). The left panel shows GFAP-GFP^+^ cells inside the hBO slice, right panel shows GFAP-GFP^+^ cells migrating away from the hBO slice. D. Epifluorescent images of whole and sliced cortical hBOs at 239 DIV (5 dpi) transduced with pAAV.GFAP.eGFP.WPRE.hGH outside of the chip, showing GFAP-GFP^+^ astrocytes (cyan). Top panels show whole hBOs, bottom panels show sliced hBOs.

First, we confirmed that freshly sliced hBO sections (unguided and cortical) could attach to the Matrigel-coated glass bottom of the chip (Fig. 3A), where they showed cell sprouting and migration (Fig. 3B and S2F). However, it was apparent that hBOs sliced post 100 DIV already contained a necrotic core (Fig. 3B). To circumvent this, we sectioned hBOs before necrotic core formation and cultured them free-floating in cell culture medium (suspension culture), allowing them to mature and develop astrocytes. However, slices sectioned multiple weeks or even days before on-chip culture did not attach to the coated glass bottom of the chip (data not shown). Therefore, we decided to continue with using only fresh slices of cortical hBOs, which generally showed less core necrosis than unguided hBOs due to their smaller size. Freshly sectioned slices of mature cortical hBOs (239 DIV) could properly attach to the glass bottom of the chip, where they showed cellular sprouting and migration (Fig. 3B). Upon sectioning or a few days later, the necrotic core was released, leaving a viable donut-like shaped hBO slice.

To determine whether the sprouting and migrating cells were astrocytes, we transduced on-chip hBO slices with an adeno-associated virus (AAV) that induces expression of green fluorescent protein (GFP) under the GFAP promoter.^41^ We confirmed that part of the cells migrating from the hBO slice expressed GFP, indicating their astrocytic identity (Fig. 3C). We also found that transducing hBOs before rather than after slicing still allowed sufficient AAV penetration for GFP expression in astrocytes located near the rim of the hBO (Fig. 3D). This approach allows for the selection of freshly sectioned slices with GFAP^+^ astrocytes on the day of co-culture initiation and was therefore used moving forward.

### hiECs form a monolayer onto the hydrogel-laden CPB contacting hiBPCs

**Fig. 4:**
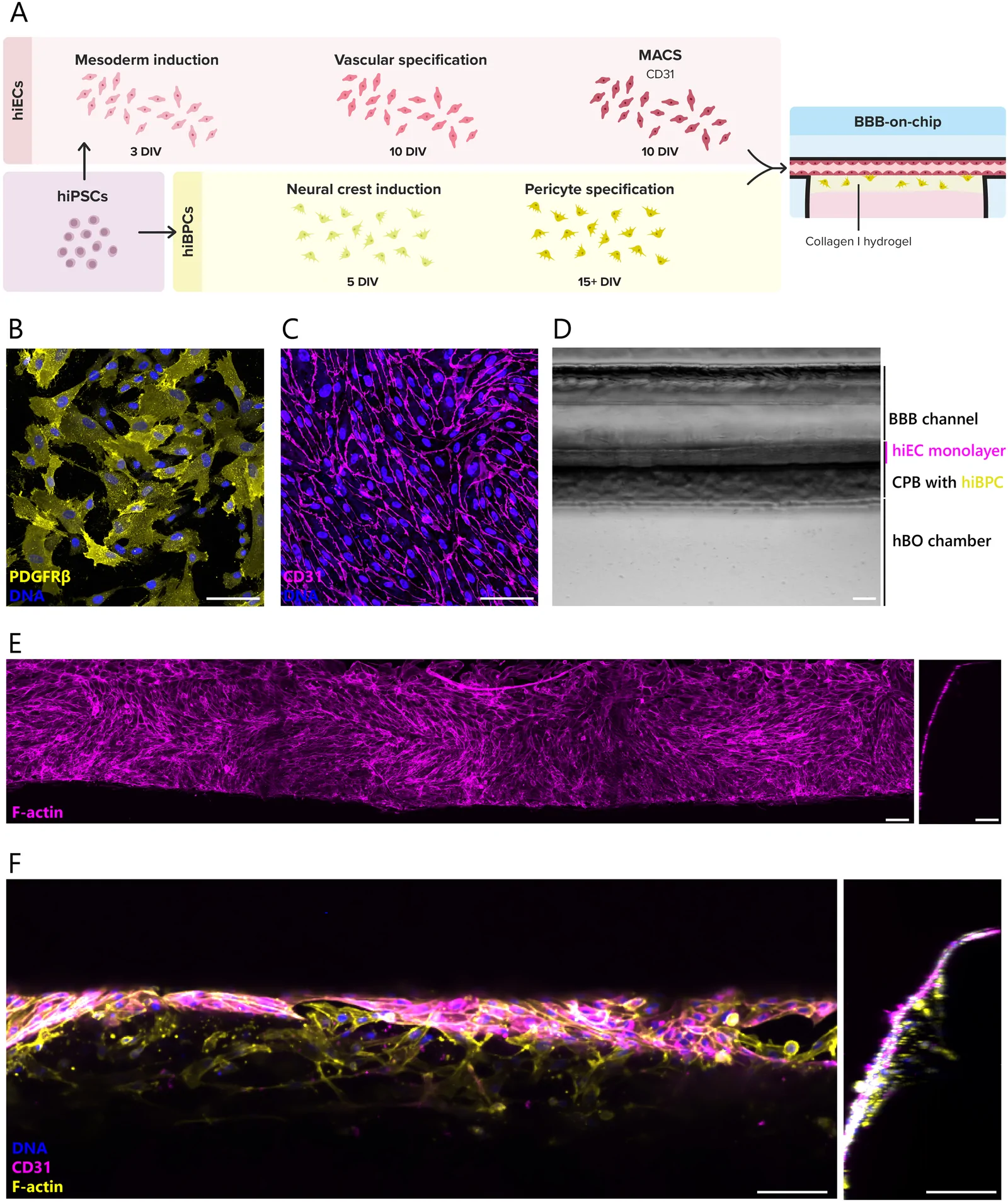
Biofabrication of the BBB compartment on the CONNECT chip. A. Schematic illustration of the CONNECT BBB-on-chip. B. Confocal image of hiBPCs stained for PDGFR-β (yellow). Nuclei are counterstained with DAPI (blue). C. Confocal image of hiECs stained for CD31 (magenta). D. Brightfield image of CONNECT chip with hiECs in BBB channel and hiBPCs in hydrogel-laden CPB. E. Maximum intensity projection (left) and *YZ* cross-section (right) of CONNECT BBB channel laden with F-actin^+^ hiECs (magenta) forming a monolayer onto the collagen gel. F. *XY* (left) and *XZ* (right) cross-sections of CONNECT CPB laden with F-actin^+^ CD31^+^ hiECs (magenta + yellow) and hydrogel-embedded F-actin^+^ CD31^−^ hiBPCs (yellow). Nuclei are counterstained with DAPI (blue). All hiBPCs and hiECs in this figure are differentiated from iPSC line 8. Scale bars: 100 μm.

We then proceeded to form a BBB vessel-like structure on-chip (Fig. 4A). For that, hiBPCs (Fig. 4B) were first encapsulated in a collagen I hydrogel and loaded into the CPB. Due to capillary forces, the gel is kept within the CPB and forms a meniscus along the *YZ* axis. After polymerization, the BBB channel was coated with collagen IV to improve hiEC adhesion. Then, hiECs (Fig. 4C) were added to the BBB inlet and adhered onto the hydrogel in the CPB. After 2 days, hiECs had formed a monolayer on the gel (Fig. 4D). At day 5, CHIR was added to the BBB channel inlets to induce BMEC specification.^29^ At day 7, chips were fixed, stained and imaged. hiECs have endothelized the gel interface entirely (Fig. 4E). hiBPCs embedded in the hydrogel migrated towards the hiECs and established physical contact points with them (Fig. 4F). We also tested sequential seeding of hiBPCs and hiECs in the BBB channel. However, hiBPCs intercalated with hiECs, which led to gaps in the endothelial monolayer (data not shown). Therefore, we proceeded with hiBPC encapsulation in the gel moving forward.

### hBO-derived astrocytes ensheathe hiBMECs and increase GFAP intensity over time

**Fig. 5:**
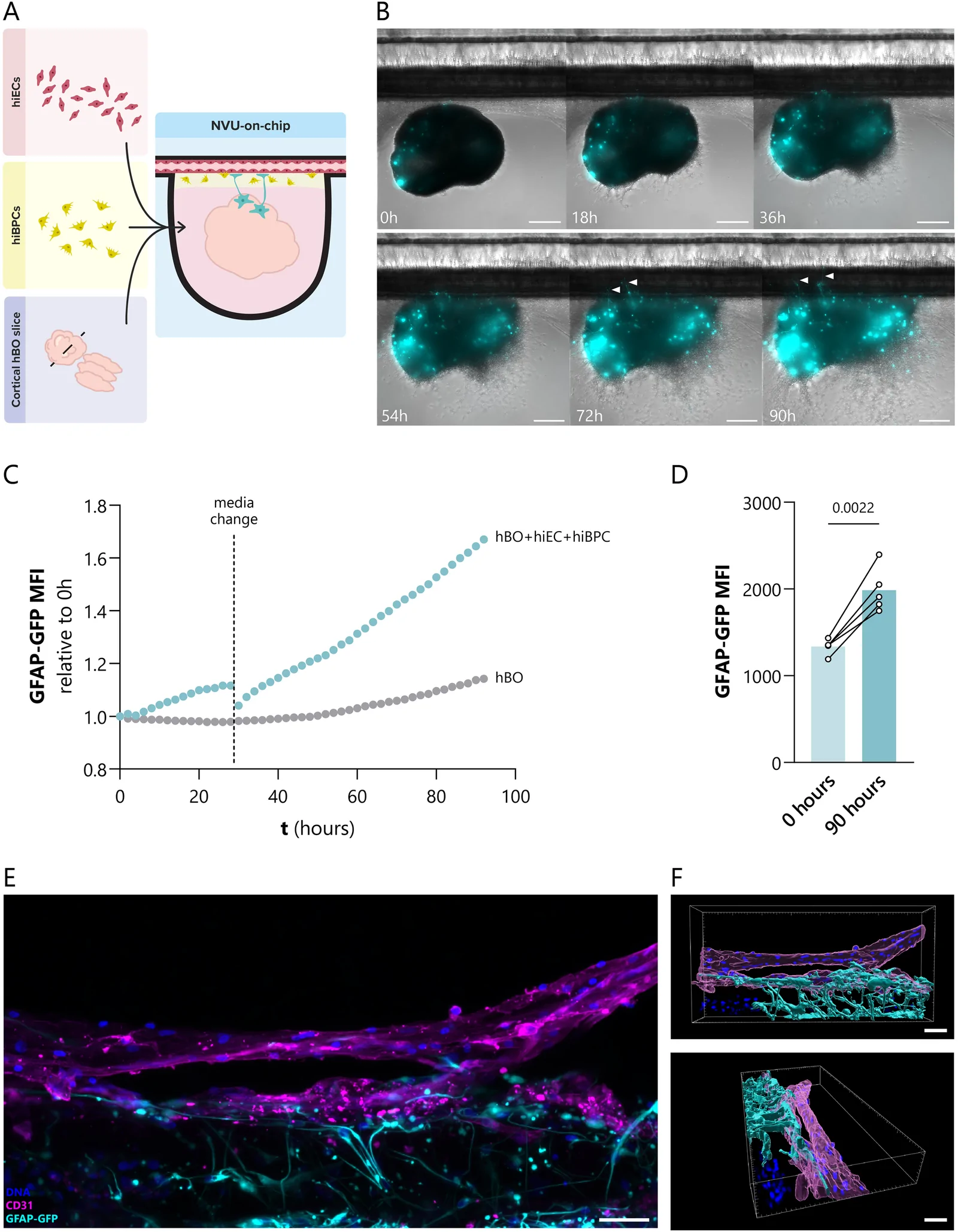
Co-culture of hiBMECs, hiBPCs, and cortical hBO slices on the CONNECT chip. A. Schematic illustration of the CONNECT NVU-on-chip. B. Time-lapse snapshots of an hBO slice infected with pAAV.GFAP.eGFP.WPRE.hGH for 48 hours and co-cultured with hiBPCs (as depicted in Fig. S3B) in the CONNECT chip for 90 hours. Scale bars: 300 μm. C. Quantification of mean fluorescence intensity of GFP signal in hBO over 90 hours of culture, normalized to initial (*t* = 0) fluorescence. In grey, hBO mono-culture; in blue, triple co-culture of hBO, hiEC and hiBPC. D. Mean fluorescence intensity of GFP signal in hBO at 0 hours (initial) and 90 hours (final) of five imaged chips (2× hiEC, 2× hiBPC, 1× hiEC + hiBPC). Two-tailed paired *t*-test. E. Immunocytochemistry of CONNECT NVU-on-chip showing direct cell–cell contact points between GFAP-GFP^+^ hBO astrocytes (cyan) and CD31^+^ hiECs (magenta). Nuclei are counterstained with DAPI (white). Scale bar: 60 μm. F. 3D reconstruction highlighting end feet-like structures from hBO astrocytes ensheathing hiBMEC monolayer. Scale bar: 60 μm.

Having defined the conditions for on-chip culture of hBO slices and hiECs/hiBPCs separately, we proceeded to optimize the timing for co-culture of all components (Fig. 5A). Since we hypothesized that hBO-derived astrocytes would need multiple days to reach the BBB channel, we first initiated on-chip culture of an hBO slice 7 days before seeding the hiECs (data not shown). However, we noticed CPB gel disintegration after 7 days in culture, possibly due to mechanical forces from medium changes or ECM remodelling by hBO-derived cells. This led to leakage of hiECs from the BBB channel to the hBO chamber upon seeding. Therefore, we decided to seed all cellular components on the same day. First, hiBPC-laden collagen I gel was loaded into the CPB, then the BBB channel was coated with collagen IV, hiECs were seeded into the BBB channel, the hBO chamber glass bottom was coated with collagen IV/laminin, and finally, one hBO slice was placed in the hBO chamber, as close as possible to the CPB.

To observe how hBO-derived astrocytes respond to the different BBB cell types, we co-cultured hBO slices with hiECs, hiBPCs, or a combination of both (Fig. S3A–C, Videos S1–S5). In the presence of hiECs and/or hiBPCs, hBO slices attached to the glass chip bottom and showed cellular sprouting and migration over the span of 90 hours (Fig. 5B, Videos S1–S5). Sprouting cells were partly of astrocytic identity as indicated by GFAP-GFP positivity. In a specific co-culture between an hBO slice and hiBPCs, multiple astrocytes were seen sprouting through the CPB and contacting the BBB channel, despite the absence of hiECs (Fig. 5B and S3B, Video S4). In some co-cultures, the entire hBO slice moved towards the hiBPCs and/or hiECs over a span of 90 hours (Videos S2, S4 and S5).

Notably, we observed a significant increase in GFAP-GFP fluorescence intensity over time in all co-cultures (Fig. 5B–D). Moreover, at the endpoint (90 hours), we performed immunocytochemistry on a triple co-culture and observed multiple hBO-derived GFAP-GFP^+^ cells establishing direct contact with hiBMECs in the BBB channel (Fig. 5E). Using 3D resconstuction, we observed GFAP-GFP^+^ endfeet-like structures ensheathing the hiBMEC monolayer at the BBB channel (Fig. 5F, Video S6), recapitulating morphological aspects of the glia limitans.

### hiBMECs attain BBB properties and align with flow direction on-chip

**Fig. 6:**
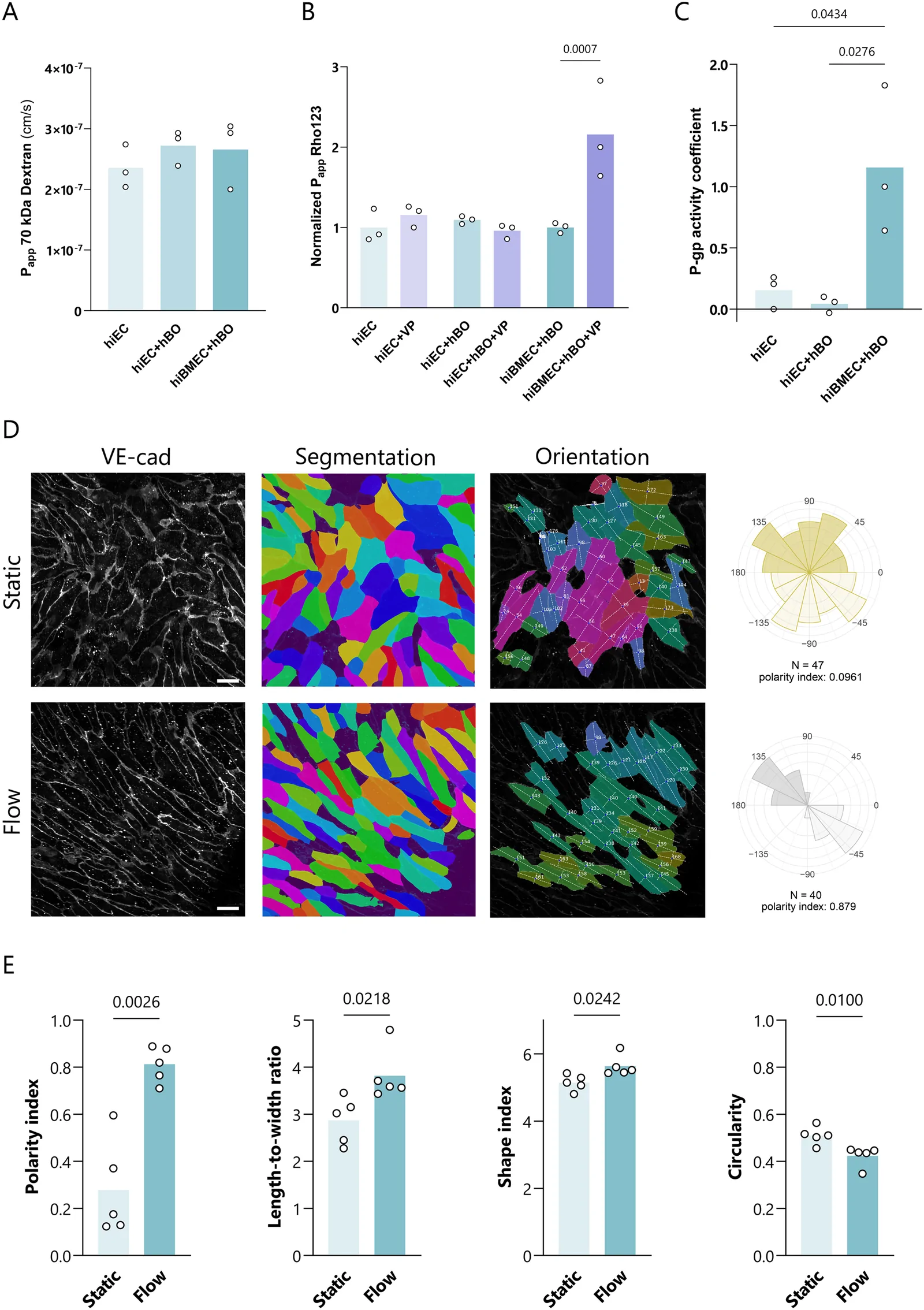
BBB properties and alignment of hiBMECs on the CONNECT chip. A. Apparent permeability (*P*_app_) to 70 kDa TRITC-dextran of hiEC, hiEC + hBO and hiBMEC + hBO. B. *P*_app_ to P-gp substrate rhodamine 123 (Rho123) of hiEC, hiEC + hBO and hiBMEC + hBO, with or without treatment with P-gp inhibitor verapamil (VP). C. P-gp activity coefficient of hiEC, hiEC + hBO and hiBMEC + hBO, based on the ratio of Rho123 *P*_app_ between VP-treated and untreated cells. Points represent individual fluidic units. D. Representative images of VE-cadherin expression (grey) in hiBMECs under static or flow conditions, resulting segmentation, alignment profiles and circular histograms of cell orientation distribution. Scale bars: 30 μm. E. Polarity index, length-to-width ratio, shape index and circularity of hiBMECs under static or flow conditions. Points represent average of all quantified cells per individual fluidic unit. Welsh's *t*-test.

To validate BBB properties on-chip, we assessed the permeability of fluorescent 70 kDa dextran across the hiEC-laded BBB channel. We observed that hiECs and hiBMECs show similarly low values of permeability, regardless of the presence of hBOs (Fig. 6A). These values were two orders of magnitude lower than chips with hiBPCs and no hiECs (Fig. S4A). Then, we evaluated the activity of P-glycoprotein (P-gp), an important BBB efflux transporter, by quantifying the permeability of rhodamine-123, a P-gp substrate, with or without the presence of verapamil, a P-gp inhibitor. We observed an increase of permeability in verapamil-treated hiBMECs but not in hiECs, with or without hBOs (Fig. 6B and C). This resulted in a P-gp activity coefficient significantly higher in hiBMECs than hiECs, indicating that P-gp was only active in hiBMECs and not in hiECs, as previously reported.^29^

Finally, we evaluated the effect of flow-induced shear stress in hiBMECs. We observed that, despite the low shear stress peak and flow bidirectionality, hiBMECs were able to align with flow direction (Fig. 6D). This was evident by an increase in polarity index, length-to-width ratio and cell shape index, and decrease in circularity (Fig. 6E and S4B and C). Interestingly, VE-cadherin expression and junctional morphology were not affected (Fig. S4D).

## 4. Discussion

In this study, we describe the design and development of a complex, 3D iPSC-based model of the NVU on a novel microfluidic chip. To our knowledge, the CONNECT chip is the first microfluidic system to combine hBO technology with a functional vessel-on-chip that does not depend on angiogenesis. The membrane-free design allows for direct interaction between an hBO and a singular, perfusable vessel-like structure that can obtain and maintain BBB properties, while the chip materials and open-top architecture are compatible with high-resolution optical imaging and downstream analytical workflows.

Our NVU-on-chip model sets itself apart from simpler systems by the incorporation of a three-dimensional, multicellular brain-like environment. Hereby, barrier properties of the vessel-like structure can be enhanced and maintained by other cell types of the NVU, including pericytes and astrocytes. Here, we show that hiBPCs induce P-gp activity in hiBMECs, validating previous reports. However, it remains elusive whether hBO-derived astrocytes have the potential to regulate water or ion homeostasis *via* endfeet channels such as aquaporin 4 (AQP4) and the inward rectifier-type potassium channel Kir4.1.^42^

*In vivo*, astrocytes directly interact with ECs to help maintain the barrier properties of the brain vasculature.^43^ Astrocytes innately develop in hBOs and can engage in physical interactions with vessel-like structures upon hBO vascularization.^44^ Here, we demonstrate that hBO-derived astrocytes extend their processes through a membrane or hydrogel and contact ECs without disrupting their monolayered organization or inducing angiogenesis. This indicates that the integrity of the vessel-like structure persists even when exposed to chemoattractant cues that the hBO may secrete, such as vascular endothelial growth factor (VEGF).^17^ Whilst VEGF promotes angiogenesis, it leads to a permeable BBB.^19,20^ Our approach does not rely on angiogenesis, which should lead to more robust barrier properties in comparison to vascularized hBOs.^10,45^ Indeed, we observed that hiBMECs display low permeability and align with flow direction on-chip, highlighting a functional BBB phenotype. Further work will incorporate tight junction markers (*e.g.* claudin-5, occludin, zonula occludens-1) for comprehensive molecular validation. Interestingly, we found that hBOs co-cultured with hiECs and/or hiBPCs showed increased fluorescence intensity levels of GFAP-GFP over time. We hypothesize that hiECs and hiBPCs promote the expression of GFAP in astrocytes by secreting factors such as leukemia inhibitory factor (LIF) and VEGF.^46–49^

The CONNECT chip design is based on the pinning effect, a physical phenomenon where an object (*i.e.*, the hydrogel) becomes immobile due to external forces (*i.e.*, the capillary pressure at the CPB). In contrast with the phaseguide technology,^50^ which also exploits the pinning effect, the placement of a curved CPB above the hydrogel space allows for the formation of a channel with a single sharp edge instead of three, which better represents the cylindrical vessel geometry and minimizes non-uniform wall shear stress profiles.^51^ Moreover, the open-top design allows for the collection of abluminal supernatant for permeability or transport assays, or the organoid itself for downstream analysis.

Despite our efforts to maximize biological complexity in our model, substantial differences from the *in vivo* BBB remain due to technical limitations that are commonly recognized in the field of BBB microfluidics.^4^ For instance, although a confluent monolayer of hiECs was grown on the hydrogel-laden CPB, we were unable to fully endothelialize the entire surface of the BBB channel. This may lead to irregular distribution of shear stress, which in turn could compromise BBB integrity.^52,53^ Moreover, on-chip vessel coverage of both astrocytes and pericytes is relatively low compared to *in vivo*,^54,55^ which may also compromise barrier properties. Cell density optimization and formal quantifications (*e.g.*, percentage of organoid slices establishing contact, migration distance, contact density) are planned for follow-up studies. Furthermore, the BBB channel is wider than the *in vivo* microvasculature (<100 μm in diameter) and the CPB dimensions are larger than surrounding basement membrane (BM),^56^ possibly resulting in sub-physiological levels of shear stress^53^ and perturbed astrocyte-EC interactions, respectively. Decreasing channel diameter, widening reservoir cross-sectional area and increasing tilt angle could lead to shear stress values closer to physiological levels. Lastly, although collagen I is commonly used in on-chip models due to its stability,^57^ it is not an accurate representation of the brain BM.^58^ An ideal hydrogel should better reflect BM composition (*i.e.*, contain collagen IV, laminin, nidogens, proteoglycans) to promote intercellular crosstalk and BBB function while still providing structural integrity.

In conclusion, the CONNECT chip represents a versatile NVU model that can serve several purposes. Our design allows for multiple aspects of drug screening to be carried out simultaneously in one co-culture unit of the chip. These include BBB crossing, drug effects on both the vasculature and the brain parenchyma, and observing which brain cell types are impacted by the drug. Screening can be performed at medium throughput, as one plate holds eight chips with five co-culture units per chip (40 units per plate). To confirm translational utility, future work will benchmark our platform against a standard panel of CNS-penetrant *versus* non-penetrant compounds. Moreover, the open-top design of the hBO chamber allows for media sampling (*e.g.*, permeability and transport assays) and easy extraction of the hBO for further analysis (*e.g.*, RNA isolation, immunohistochemistry, and omics). Furthermore, NVU formation in the CONNECT system can be monitored in real time using live imaging, enabling the investigation of fundamental biological questions (*e.g.*, the initiation of EC-astrocyte interactions in development). Lastly, the CONNECT model is fully iPSC-based, providing a platform for disease modeling and personalized medicine using patient-derived cells.

## Author contributions

H. Nogueira Pinto, L. Kistemaker: conceptualization, methodology, formal analysis, investigation, writing – original draft, visualization. M. Ponomarenko: conceptualization, design & prototyping, writing – review & editing. A. A. Ermakov: conceptualization, methodology, investigation. M. Karsten-van Diepen, S. M. A. van der Pol: investigation. R. J. Pasterkamp: resources. S. D. Beekhuis-Hoekstra, N. M. de Wit: methodology, supervision. E. J. van Bodegraven: conceptualization, writing – review & editing, supervision. H. E. de Vries, E. M. Hol: conceptualization, writing – review & editing, supervision, funding acquisition.

## Conflicts of interest

There are no conflicts of interest to declare.

## Supplementary Material

LC-026-D6LC00157B-s001

LC-026-D6LC00157B-s002

LC-026-D6LC00157B-s003

LC-026-D6LC00157B-s004

LC-026-D6LC00157B-s005

LC-026-D6LC00157B-s006

LC-026-D6LC00157B-s007

## Data Availability

We would like to inform you that the data supporting this article have been included as part of the supplementary information (SI) (Fig. S1–S3, Tables S1 and S2, Videos S1–S5). Supplementary information is available. See DOI: https://doi.org/10.1039/d6lc00157b.
