# Supplementary material for: A human iPSC-based perfusable and membrane-free neurovascular unit-on-chip connecting brain organoids to the blood–brain barrier
Source: Lab Chip. 2026 Sep 11;26(19):5198–215. doi: 10.1039/d6lc00157b (PMC13588194; doi:10.1039/d6lc00157b)
Supplement: Supplementary file 1 [file LC-026-D6LC00157B-s001.pdf]

Supplementary Information (SI) for Lab on a Chip.
This journal is © The Royal Society of Chemistry 2026
Supplementary Information (SI) for Lab on a Chip.
This journal is © The Royal Society of Chemistry

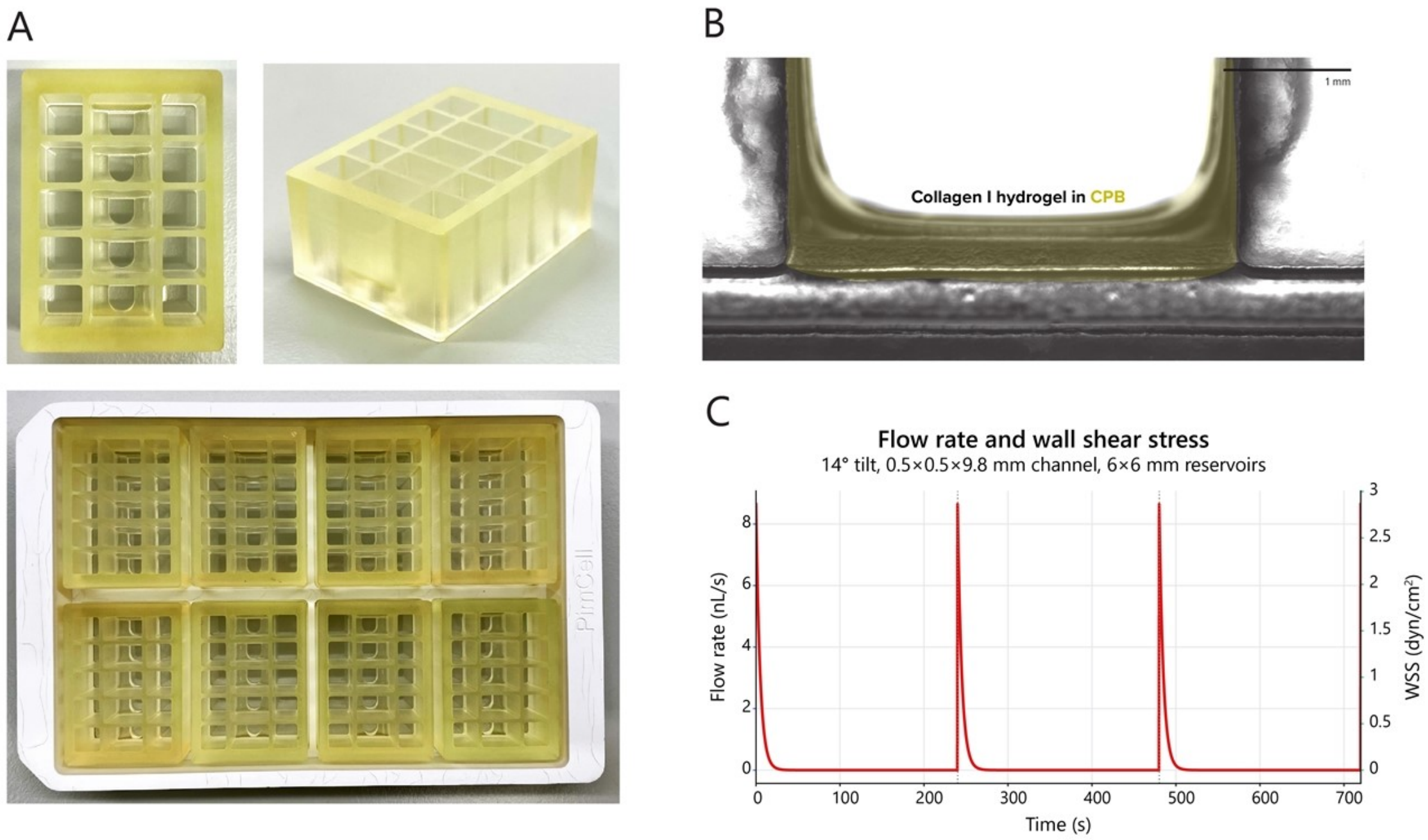

Supplementary Figure 1. Pictures and flow simulation of the CONNECT chip.

A. One chip contains 5 co-culture units. Left: top view. Right: side view. A standard well-plate-sized holder holds eight chips. **B.** Brightfield image of the CONNECT chip with collagen I hydrogel-laden CPB (yellow color indicates hydrogel placement). **C.** Flow rate and wall shear stress simulation in BBB channel over a period of 12 minutes.

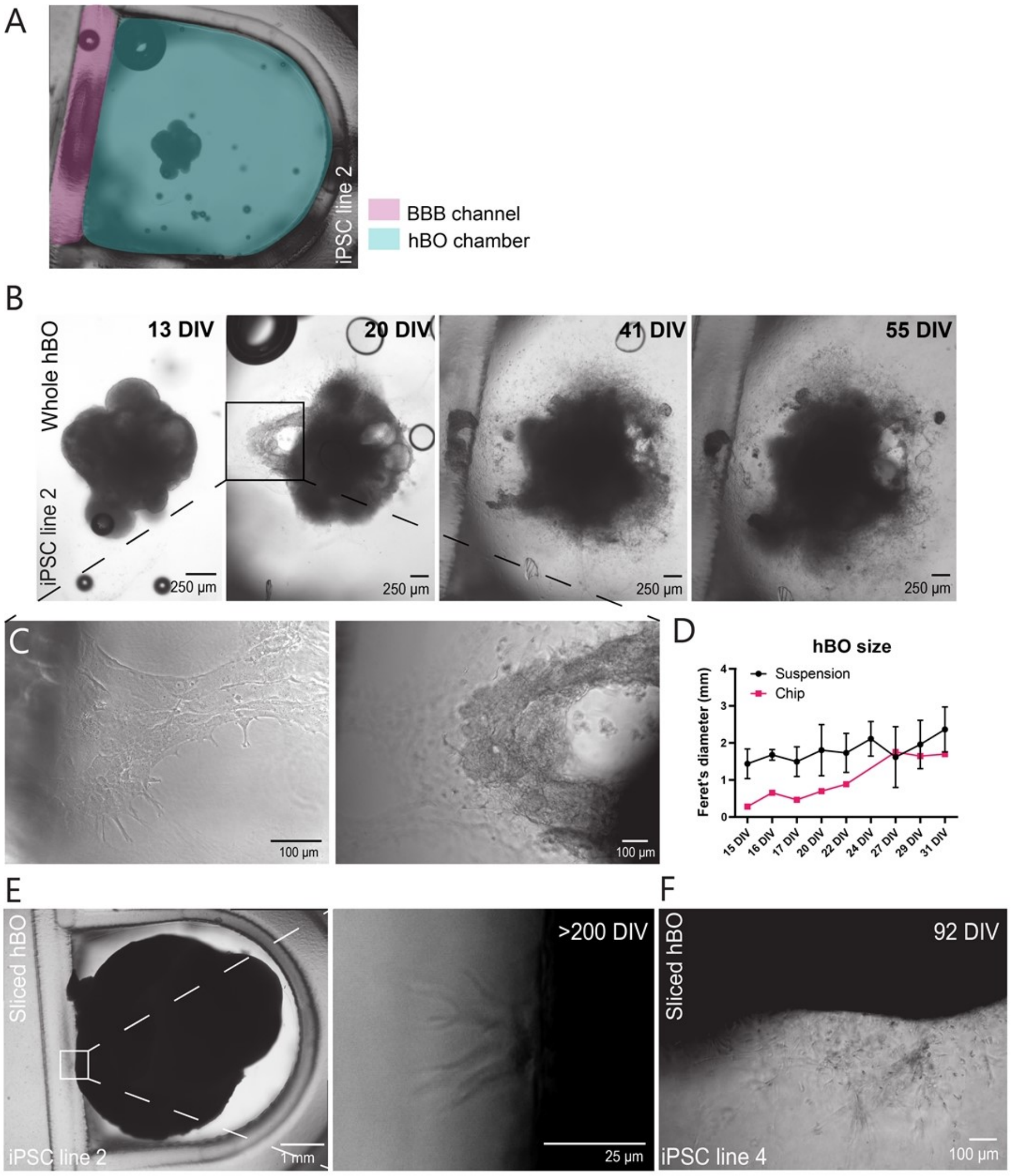

Supplementary Figure 2. Culturing whole and sliced unguided hBOs in the CONNECT chip prototype.

A. Brightfield image of an unguided hBO at 13 DIV (iPSC line 2) embedded in Matrigel in the CONNECT chip prototype. Pink color indicates the BBB channel, whereas blue color indicates the hBO chamber. **B**. Brightfield images of unguided hBO (iPSC line 2) growth on the CONNECT chip prototype. From left to right: 13 DIV, 20 DIV, 41 DIV, and 55 DIV. **C**. Brightfield images of unguided hBO-derived cell migration on the CONNECT chip prototype. Left panel: cells sprouting/migrating and contacting the CPB. Right panel: cells sprouting/migrating from the hBO. **D**. Size (Feret's diameter) of unguided hBOs (iPSC line 2) on the CONNECT chip prototype versus in suspension culture. N=1 (on-chip) and N=6-16 individual hBOs (suspension). Datapoints represent group mean, and error bars indicate standard deviation. *No statistical testing was performed.* **E**. Left panel: brightfield image of an unguided hBO (iPSC line 2) at 200+ DIV (previously cultured in suspension) embedded in Matrigel on the CONNECT chip prototype. In the right panel, a cluster of cells can be seen sprouting into the Matrigel-laden CPB. **F**. Brightfield image of unguided hBO slices of iPSC line 4 at 92 DIV placed on the Matrigel-coated glass bottom of the CONNECT chip prototype.

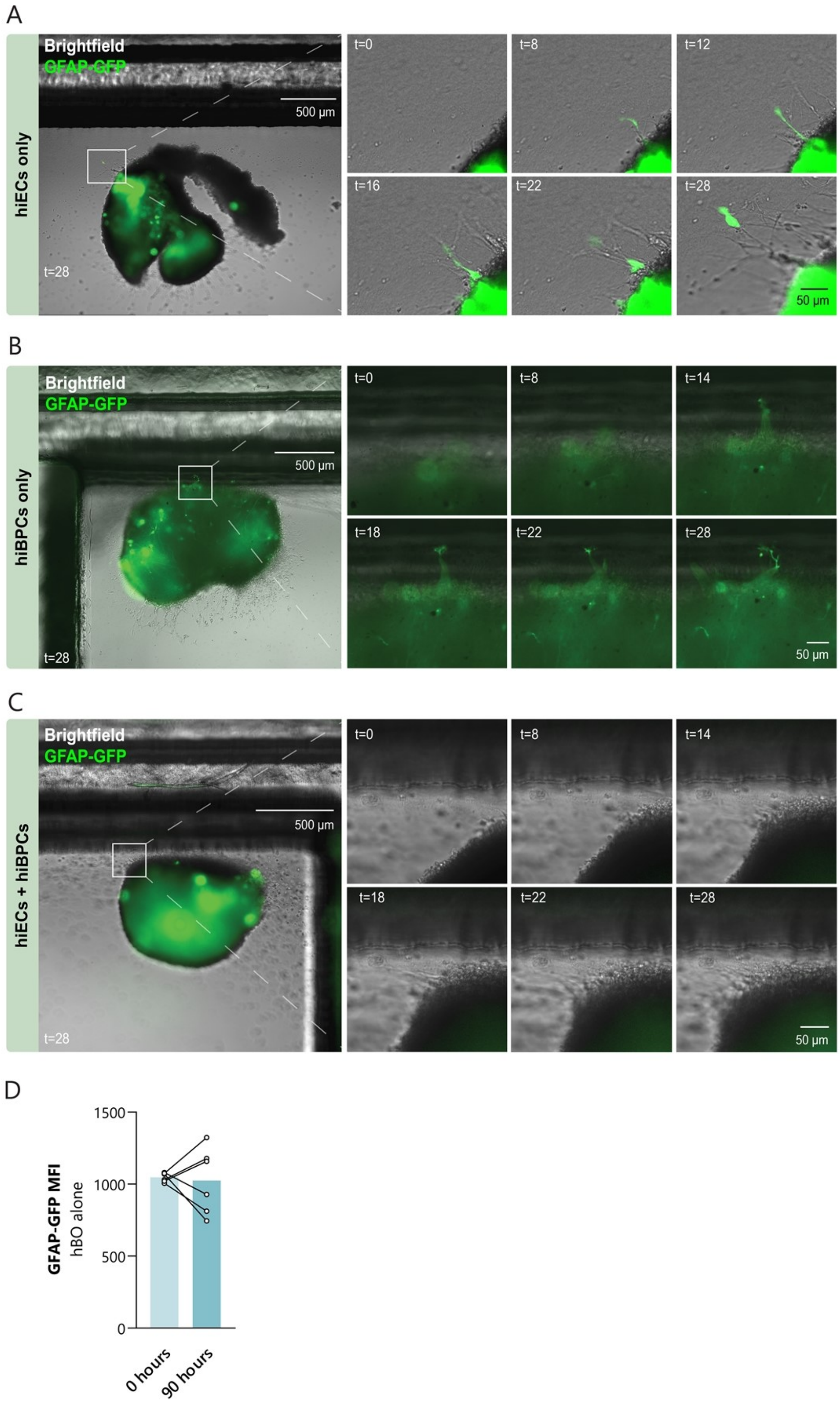

Supplementary Figure 3. Live imaging of GFAP-GFP-transduced hBO slices co-cultured with hiECs and/or hiBPCs on the CONNECT chip.

A-C. Merged brightfield and epifluorescent snapshots of live imaged co-cultures of hBO slices with hiECs only (**A**), hiBPCs only (**B**), or both (**C**) over a span of 28 hours. Left panels show the whole co-culture at t=28, smaller panels on the right show highlighted regions of interest (ROIs) over time. **D.** Mean fluorescence intensity of GFP signal in hBO at 0 hours (initial) and 90 hours (final) of six imaged chips of hBO slice monoculture.

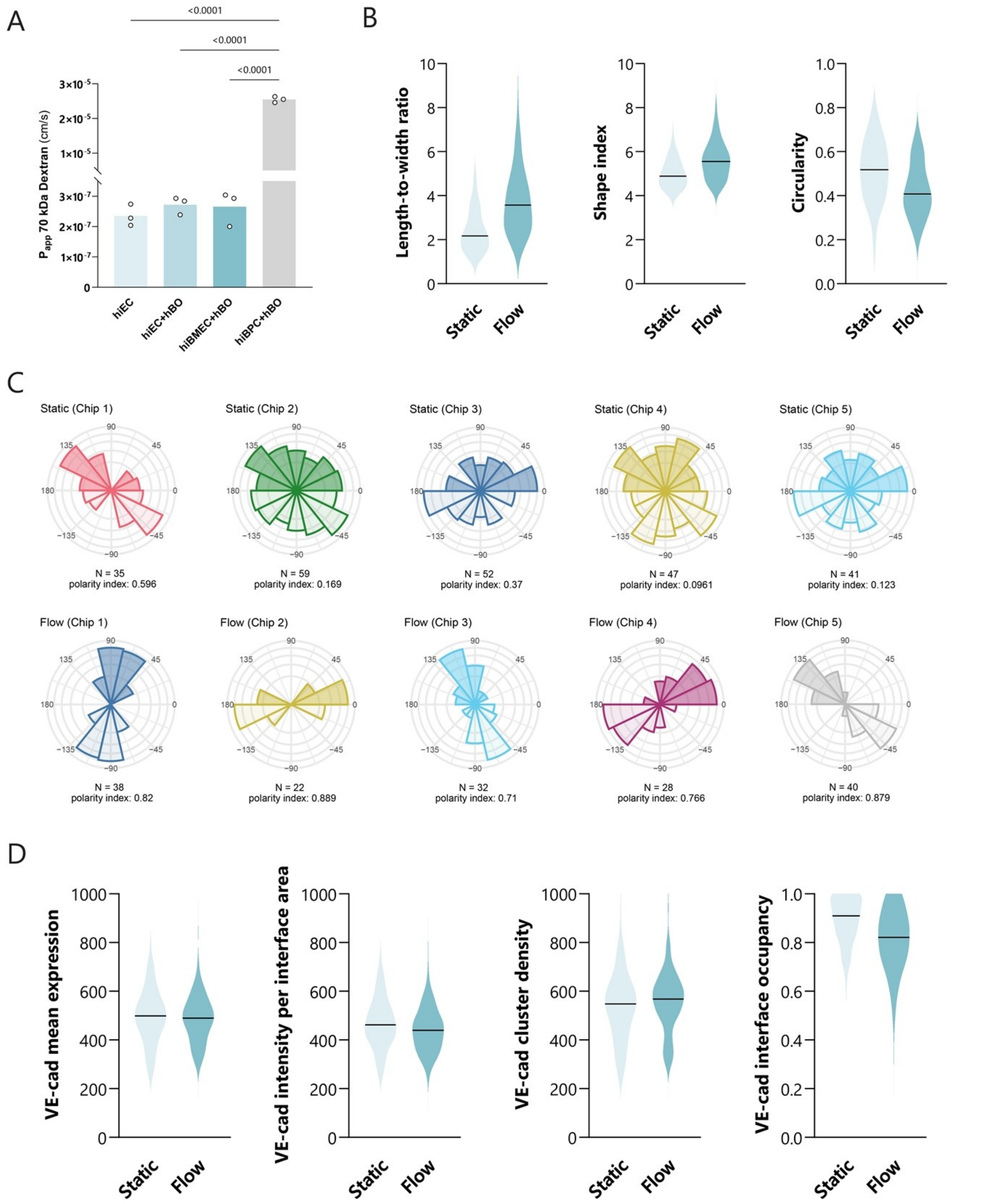

Supplementary Figure 4. Extended data on permeability and alignment of hiBMECs on the CONNECT chip. A. Apparent permeability (P_{app}) to 70 kDa TRITC-dextran of hiEC, hiEC+hBO and hiBMEC+hBO compared to hiBPC+hBO. Points represent individual fluidic units. Two-tailed Student's t-test. **B.** Per-cell distribution of length-to-width ratio, shape index and circularity of hiBMECs under static or flow conditions. **C.** Circular histograms, number of cells and polarity indices of all analysed hiBMECs in each fluidic units (5 fluidic units per condition). **D.** Per-cell distribution of VE-caherin mean expression, indensity per interface area, cluster density and interface occupancy in hiBMECs under static or flow conditions.

Supplementary Table 1. Composition of media used for hBO (slice) culturing.

Medium	Composition
**HES0**	DMEM-F12 (Gibco, 11320074)<br>20% KOSR (Gibco, 10828028)<br>3% FBS (Sigma-Aldrich, F7524)<br>2 mM L-Glutamine (L-Glu; Gibco, 25030024)<br>1 × MEM-NEAA (Gibco, 11140035)<br>3.5 µl/100 mM ß-mercaptoethanol (Merck, 805740) in DMEM/F-12
**HES4+**	*HES0 supplemented with:*<br>4 ng/mL bFGF (Pepro-Tech, 100-18B)<br>50 µM Y-27632 dihydrochloride (Axon Medchem, 1683)
**NIM**	DMEM-F12<br>1 × N2 supplement (Gibco, 17502001)<br>2 mM L-Glu<br>1 × MEM-NEAA<br>1µg/mL heparin (Sigma-Aldrich, H3149)
**B27-**	1:1 DMEM-F12/Neurobasal medium (Gibco 21103,049)<br>100 U/mL penicillin/streptomycin (p/s; Gibco, 15140122)<br>2 mM L-Glu<br>1 × B27 supplement minus Vit-A (Gibco, 12587010)<br>0.5 × MEM-NEAA<br>0.5 × N2 supplement<br>0.025% human insulin (Sigma, I9278)<br>50 mM ß-mercaptoethanol
**B27+**	1:1 DMEM-F12/Neurobasal medium (Gibco 21103,049)<br>100 U/mL p/s<br>2 mM L-Glu<br>1 × B27 supplement plus Vit-A (Gibco, A3582801)<br>0.5 × MEM-NEAA<br>0.5 × N2 supplement<br>0.025% human insulin<br>50 mM ß-mercaptoethanol
**Neural**	Neurobasal medium<br>100 U/mL p/s<br>2 mM L-Glu<br>1 × B27 supplement minus Vit-A (Gibco, 12587010)
**Recovery**	High-glucose DMEM with L-Glutamine (Gibco, 41965039)<br>1 × penicillin-streptomycin-amphotericin B suspension (p/s/a; Sigma-Aldrich, A5955) added fresh every day<br>10% FBS<br>0.5% glucose
**ALICO**	Neurobasal medium<br>1 × p/s/a added fresh every day<br>2 mM L-Glu<br>1 × B27 supplement plus Vit-A<br>0.5% glucose

Supplementary Table 2. Composition of solutions for FUnGI tissue clearing.

Solution	Composition
**Washing buffer 1 (WB1)**	1x PBS<br>0.2% Tween-20 (Sigma-Aldrich, P1379)<br>0.2% SDS (Sigma-Aldrich, 822050)<br>0.2% Triton X-100 (Merck, T8787)<br>0.2% Bovine Serum Albumin (BSA; Merck, A7906)
**Washing Buffer 2 (WB2)**	1x PBS<br>0.2% SDS<br>2 mL Triton X-100 (Merck, T8787)<br>2 g BSA (Merck, A7906)
**Fructose, urea, and glycerol for imaging (FUnGI) clearing agent**	50% (v/v) glycerol<br>9.4% (v/v) dH_2O<br>10.6 mM Tris (Sigma-Aldrich, TRIS-RO) buffer in 3-3.2% HCl (v/v), pH 8<br>1.1 mM EDTA (Sigma-Aldrich, E5134) in dH_2O, pH 8<br>2.5 mM fructose (Sigma-Aldrich, F0127)<br>2.5 mM urea (Sigma-Aldrich, U5128)

Supplementary Table 3. A list of primary antibodies and dyes used for staining.

Primary antibody or dye	Species	Vendor	Catalog number	Dilution
**GFAP**	Rabbit	Dako	Z0334	1:1000
**CD31**	Mouse	Dako	M0823	1:100
**GFP**	Chicken	AvesLabs	GFP-1020	1:1000
**VE-cadherin**	Mouse	Santa Cruz	sc-9989	1:400
**Phalloidin AF 647**	-	Molecular Probes	A30107	1:400

Supplementary Table 4. A list of secondary antibodies used for staining.

Secondary antibody	Vendor	Catalog number	Dilution
**Donkey anti-Mouse AF594**	Jackson ImmunoResearch	AB_2340854	1:250
**Donkey anti-Rabbit AF488**	Jackson ImmunoResearch	AB_2313584	1:250
**Donkey anti-Chicken AF488**	Jackson ImmunoResearch	AB_2340376	1:400
**Donkey anti-Mouse AF647**	Molecular Probes	A31571	1:400
